# Antibody-Proteolysis Targeting Chimera Conjugate Enables Selective Degradation of Receptor-Interacting Serine/Threonine-Protein Kinase 2 in HER2+ Cell Lines

**DOI:** 10.1021/acs.bioconjchem.3c00366

**Published:** 2023-11-02

**Authors:** Karina Chan, Preethi Soundarya Sathyamurthi, Markus A. Queisser, Michael Mullin, Harry Shrives, Diane M. Coe, Glenn A. Burley

**Affiliations:** † GSK, Gunnels Wood Road, Stevenage, Hertfordshire SG1 2NY, United Kingdom; ‡ Department of Pure and Applied Chemistry, University of Strathclyde, Glasgow G1 1XL, United Kingdom

## Abstract

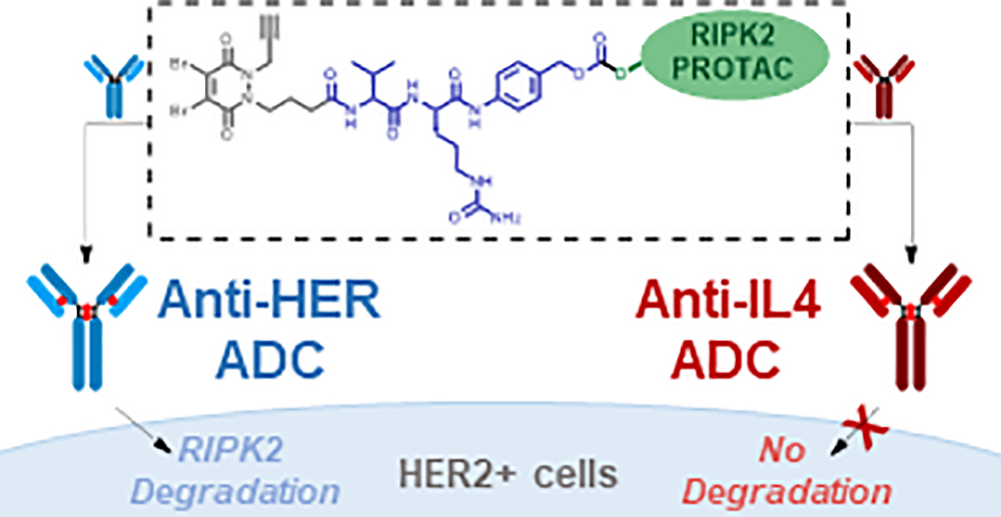

Proteolysis targeting chimeras (PROTACs) are a family of heterobifunctional molecules that are now realizing their promise as a therapeutic strategy for targeted protein degradation. However, one limitation of existing designs is the lack of cell-selective targeting of the protein degrading payload. This manuscript reports a cell-targeted approach to degrade receptor-interacting serine/threonine-protein kinase 2 (RIPK2) in HER2+ cell lines. An antibody-PROTAC conjugate is prepared containing a protease-cleavable linkage between the antibody and the corresponding degrader. Potent RIPK2 degradation is observed in HER2+ cell lines, whereas an equivalent anti-IL4 antibody-PROTAC conjugate shows no degradation at therapeutically relevant concentrations. No RIPK2 degradation was observed in HER2– cell lines for both bioconjugates. This work demonstrates the potential for the cell-selective delivery of PROTAC scaffolds by engaging with signature extracellular proteins expressed on the surface of particular cell types.

Proteolysis targeting chimeras (PROTACs) are heterobifunctional molecules that selectively degrade a protein of interest (POI).^1,2^ The mechanism of action (MoA) of PROTACs proceeds via the formation of a ternary complex with a POI and an E3 ligase, which then induces a proximity-induced ubiquitination of the POI on a surface lysine and subsequent degradation by the ubiquitin–proteasome pathway.^3,4^ A hallmark of these protein degraders is the catalytic nature of degradation,^5^ which enables recycling of the PROTAC after dissociation from the ternary complex. This unique MoA results in a longer-lasting pharmacological effect relative to conventional noncovalent inhibition,^6^ enabling lower dosages for their application *in vivo.*(7−9) A further advantage of PROTACs over conventional inhibitor strategies is the need to engage the POI ultimately for degradation rather than a modulation of protein function by stoichiometric interaction with a small molecule.^10^

At present, one major limitation of the application of PROTACs is their lack of cell selectivity and variable levels of cell permeability,^11^ which is reflected in their suboptimal pharmacokinetic properties.^12,13^ Incorporating a cell-targeting module into PROTAC designs has the potential to deliver the PROTAC cargo to the desirable cell type(s) and subsequently minimize off-target toxicity (Figure 1A).

**Figure 1:**
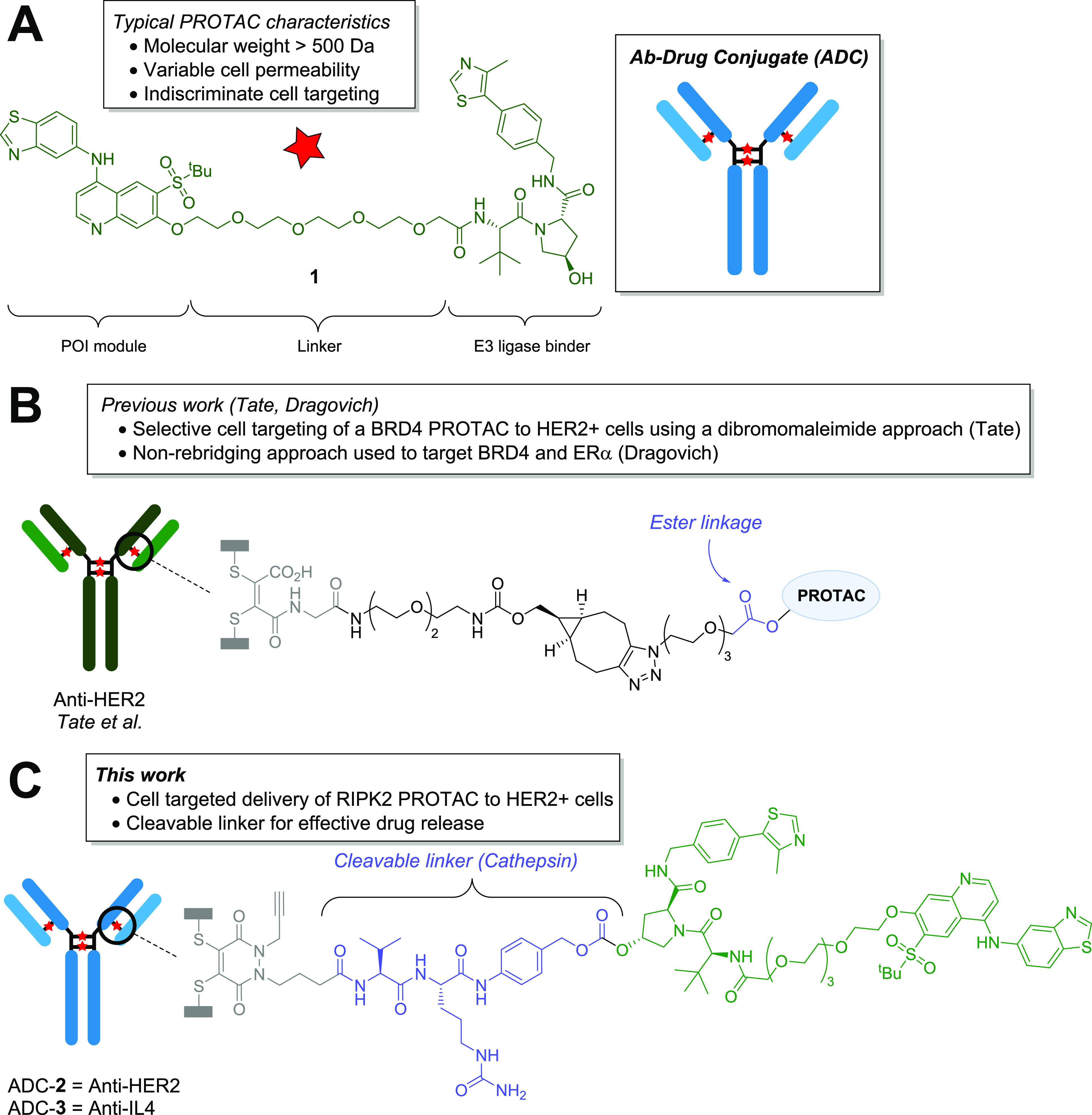
(A) General structure and characteristics of a PROTAC and ADCs. (B) Exemplar development of Ab-PROTAC conjugates. (C) Our approach: RIP2K degrading Ab-PROTAC conjugates incorporating a cleavable linkage. Gray: dibromopyridazinedione (diBrPD) conjugation motif; blue: VC-PAB linker; green: RIPK2 PROTAC. Red star indicates payload.

An emerging platform for the cell-selective delivery of PROTACs is their conjugation to an antibody (Ab).^14^ Ab-drug conjugates (ADCs) combine the ability to selectively deliver a molecular payload, such as a PROTAC, to specific cell types, thereby bypassing the need for extensive optimization of the cell uptake properties to the PROTAC scaffold (Figure 1B). While Ab-PROTACs have been developed for the cell-selective degradation of BRD4 and ERα,^15−17^ the impact of how the linkage chemistry (i.e., cleavable vs noncleavable), drug accumulation into a target cell type, and the diversity of POI can be targeted by Ab conjugation is still in its infancy. Herein, we expand the scope of Ab-PROTAC conjugates by demonstrating the cell-selective and targeted degradation of serine- and threonine-protein kinase 2 (RIPK2) in HER2+ cell lines (Figure 1C).

We selected a RIPK2 PROTAC **1**(4) and an anti-HER2 monoclonal antibody (mAb), trastuzumab,^18^ as our model system to demonstrate selective RIPK2 degradation in HER2+ cells only. Dysregulation of RIPK2-mediated pathways is associated with inflammatory bowel disease,^19^ severe pulmonary sarcoidosis,^20^ multiple sclerosis,^21^ and cancer.^22^ We hypothesized that the ability to degrade RIPK2 only in cells that express cancer biomarkers would provide the basis for cell-selective targeting.

Our design approach involved covalently linking a RIPK2 PROTAC to each Ab scaffold via a disulfide rebridging reagent (dibromopyridazinedione, diBrPD).^23−25^ This approach enabled attachment of the PROTAC linkage to a precise site on the Ab scaffold, i.e., at the interchain cysteines.^26^ The diBrPD warhead was coupled to the PROTAC via a protease cleavable valine-citrulline-*para*-aminobenzyl-alcohol (VC-PAB) linker.^27^ A second antibody, anti-IL-4 pascolizumab, was also selected as a negative control.^28^ A terminal alkyne was incorporated onto the second nitrogen of the diBrPD to act as a flexible handle for potential downstream functionalization.

The RIPK2 PROTAC **1** was attached to the VC-PAB **S6** via a carbonate linkage, which was then linked to the diBrPD by an amide bond (Scheme S3). Conjugation to the anti-HER2 mAb, trastuzumab, was achieved by reduction of the interchain disulfides by TCEP followed by addition of the diBrPD rebridging reagent **S10** to form conjugate ADC-**2**, which identified a drug-to-Ab ratio (DAR) of 4.0 (Figure 2). These exist as an interchain bridged species and an intrachain “half-body” (HB) species, where the cysteines have bridged within a single heavy chain. The control anti-IL-4 ADC-**3** was synthesized in a similar manner, which resulted in a DAR of 3.7.

**Figure 2:**
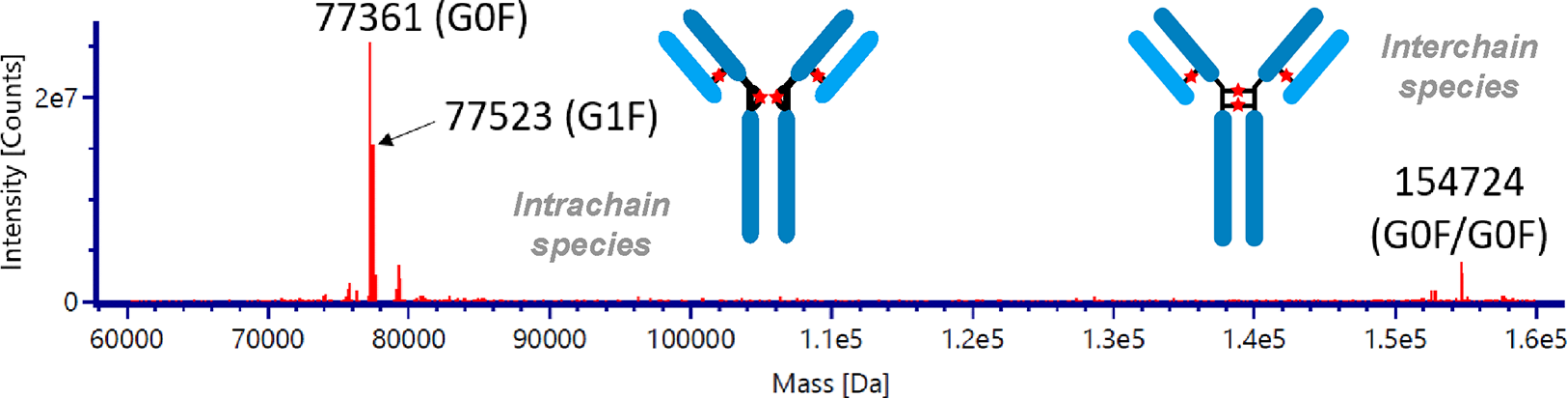
Deconvoluted mass spectrum of anti-HER2 ADC-**2**. Unmodified mAb = 147,990 Da. Calculated DAR 4 = 154,714 Da, found 154,724 (error 10 Da). Calculated half-body (HB) DAR 2 = 77,357 Da; found 77,361 (error 4 Da). **G0F** and **G1F** correspond to glycan modifications on the mAb.^29^ Red stars indicate payload.

RIPK2 degradation using ADC-**2** and ADC-**3** was assessed in a SKOV3 HER2+ ovarian cancer cell line. The anti-HER2 ADC-**2** showed similar levels of RIPK2 degradation compared to the parent PROTAC, whereas the anti-IL-4 ADC-**3** showed no degradation at 10 nM. An unexpected observation was RIPK2 degradation using ADC-**3** at concentrations above 100 nM (Figure 3A and quantification in Figure S1). As the SKOV3 cells do not have membrane-bound IL-4, we rationalized that the observed degradation might be due to nonspecific uptake mechanisms, such as macropinocytosis.^30^

**Figure 3:**
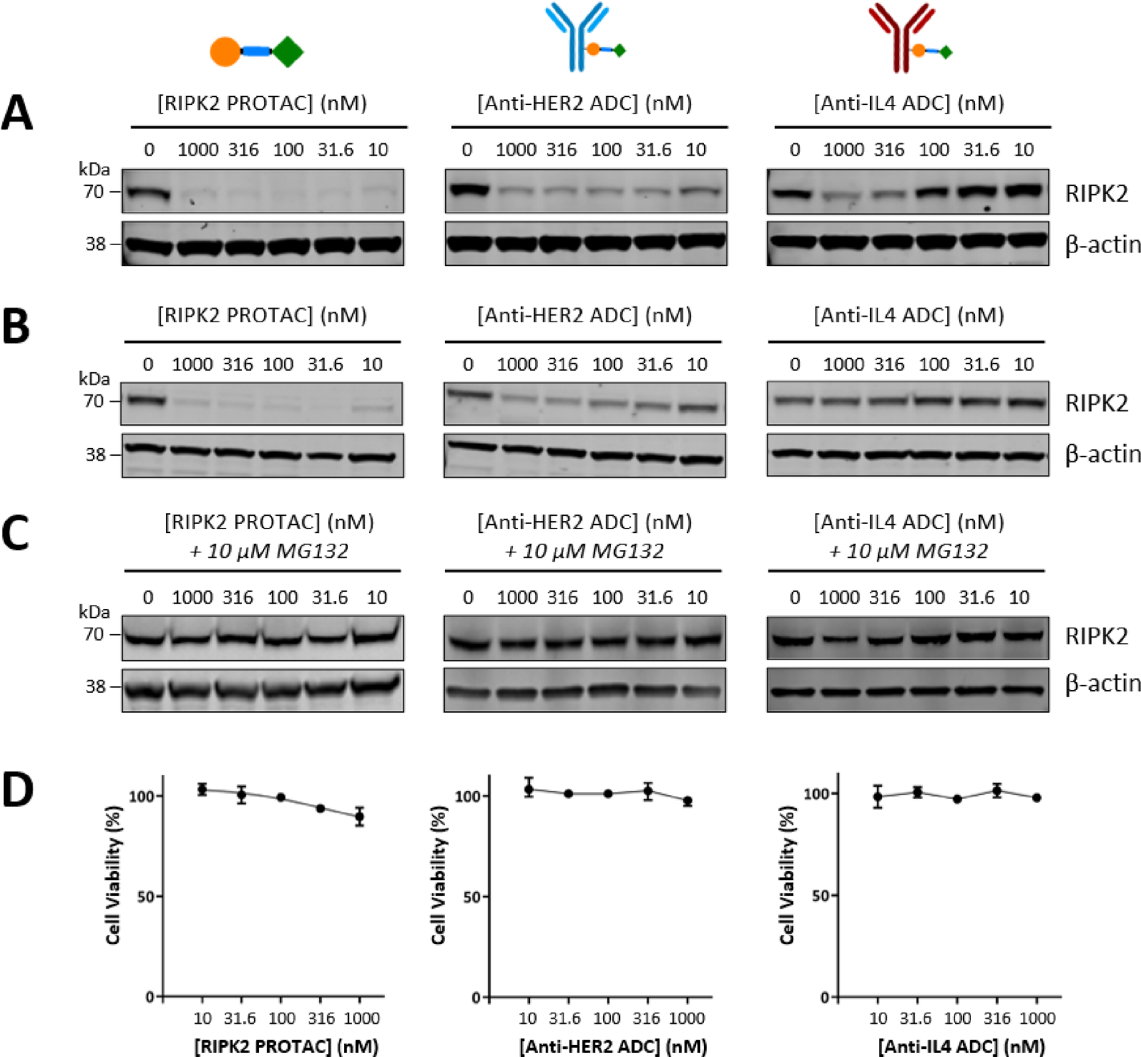
RIPK2 degradation of PROTAC **1**, ADC-**2**, and ADC-**3** in SKOV3 cells. (A) Western blot analysis after 16 h of incubation. (B) Western blot analysis after 6 h of incubation. (C) Western blot analysis after a 1 h pretreatment with 10 μM MG132 followed by a 16 h cotreatment with PROTAC **1**, ADC-**2**, or ADC-**3**. (D) CellTiter-Glo cell viability assay carried out in SKOV3 cells following a 16 h incubation with PROTAC **1**, ADC-**2**, or ADC-**3** (mean ±95% CI, *n* = 3). Concentrations shown indicate the concentration of the drug following DAR normalization for the ADCs.

Shortening of the incubation time (6 h) resulted in less RIPK2 degradation by ADC-**2** compared to that of PROTAC **1** alone (Figure 3B and quantification in Figure S2). We surmise that this is due to the uptake and release of the PROTAC from the conjugate slowing down the initial rate of degradation. To confirm that RIPK2 degradation occurred via the ubiquitin–proteasome pathway, SKOV3 cells were treated with PROTAC **1**, ADC-**2**, or ADC-**3** in the presence of 10 μM MG132, a known proteasome inhibitor.^31^ No degradation was observed for all compounds, confirming that degradation occurs via a proteasome-dependent pathway (Figure 3C). No cytotoxicity was observed up to 1 μM for both ADC-**1** and ADC-**2** (Figure 3D). To rule out the instability of the linker causing premature release of PROTAC, carbonate **S8** was subjected to conditions similar to those of the cellular assays, with minimal PROTAC release observed after a 16 h incubation at 37 °C (Figure S6). Intact MS analysis of ADC-**2** after 275 days of storage in pH 7.4 PBS at 4 °C also revealed no degradation of the conjugate (Figure S7).

Cell-selective targeting of ADC-**2** and ADC-**3** was then tested in a HEK293 HER2– cell line using a HiBiT assay. Both ADC-**2** and ADC-**3** exhibited RIPK2 degradation at higher concentrations, with a more prominent effect as the concentration exceeded 100 nM. This agrees with the fact that the similar degradation observed in SKOV3 cells is likely due to nonspecific uptake. Most importantly, no degradation was observed at 10 nM for both conjugates compared to 50% degradation when PROTAC **1** was used (Figure 4A). Again, no cytotoxicity was observed for all compounds (Figure 4B).

**Figure 4:**
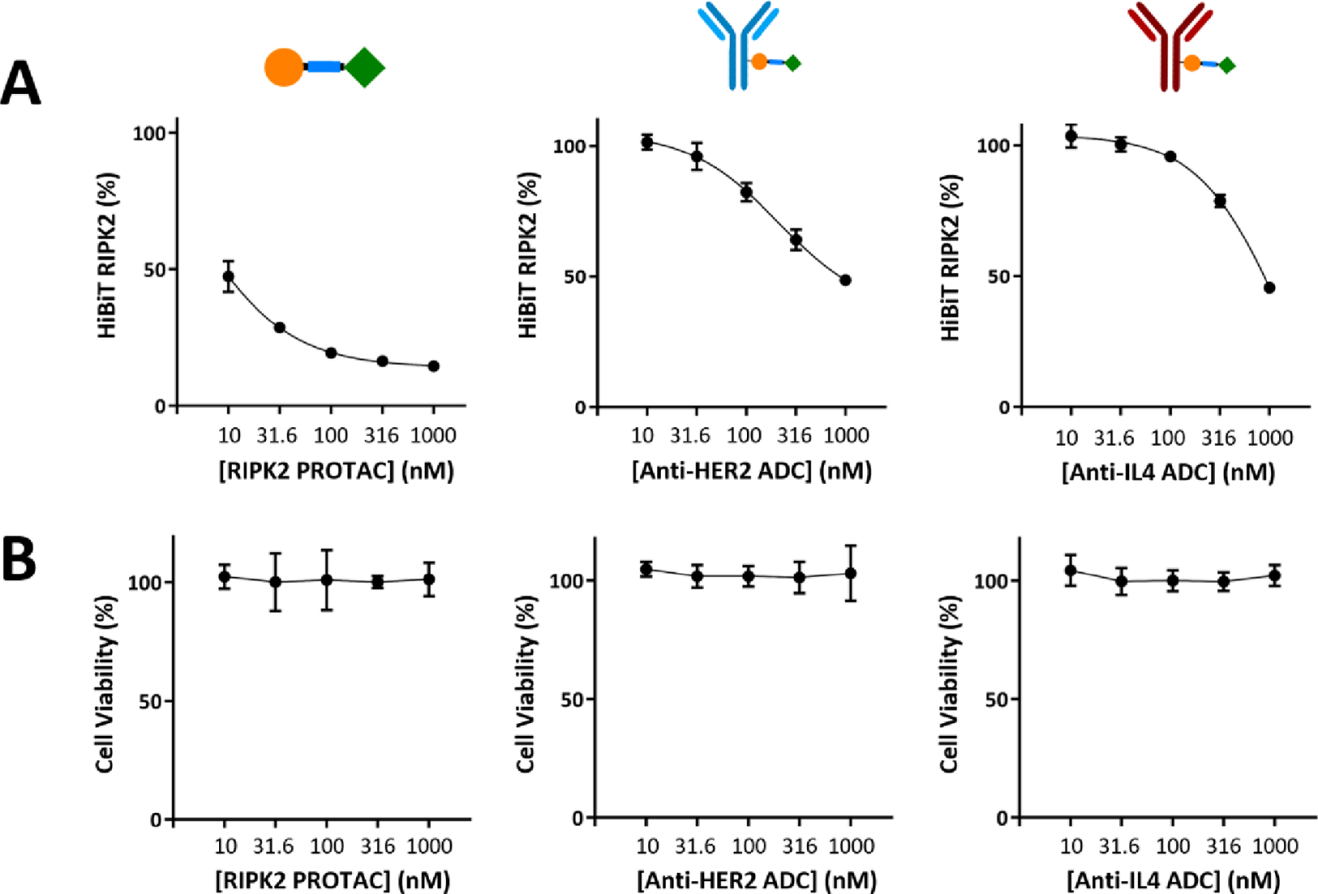
(A) RIPK2 levels in a RIPK2 HiBiT HEK293 cell line after a 16 h incubation with PROTAC **1**, ADC-**2**, or ADC-**3**. RIPK2 levels determined using the Promega Nano-Glo HiBiT Lytic detection system (mean ±95% CI, *n* = 3). (B) CellTiter-Glo cell viability assay carried out in HEK293 cells following a 16 h incubation with PROTAC **1**, ADC-**2**, or ADC-**3** (mean ±95% CI, *n* = 3). Concentrations shown indicate the concentration of the drug following DAR normalization for the ADCs.

In summary, we have demonstrated the cell-selective degradation of RIPK2 in HER2+ SKOV3 cells using an Ab-PROTAC conjugate. This approach complements ADC developments and provides a design strategy to use PROTACs, which have suboptimal physicochemical properties or where cell-selective delivery of the PROTAC payload is required.

## Supporting Information Available

Experimental procedures and compound analysis by LCMS and NMR spectroscopy, conjugation and assay methods, and additional data analysis (PDF)
