# Supplementary material for: Antibody-Proteolysis Targeting Chimera Conjugate Enables Selective Degradation of Receptor-Interacting Serine/Threonine-Protein Kinase 2 in HER2+ Cell Lines
Source: Bioconjug Chem. 2023 Nov 2;34(11):2049–54. doi: 10.1021/acs.bioconjchem.3c00366 (PMC10655034; doi:10.1021/acs.bioconjchem.3c00366)
Supplement: Supplementary file 1 — bc3c00366_si_001.pdf [file bc3c00366_si_001.pdf]

Supporting information for:

Antibody-PROTAC Conjugate Enables Selective Degradation of Receptor-Interacting Serine/Threonine-Protein Kinase 2 (RIPK2) in HER2+ Cell Lines

Karina Chan[a,b], Preethi Soundarya Sathyamurthi[a], Markus A. Queisser[a], Michael Mullin[a], Harry Shrives[a], Diane M. Coe[a], Glenn A. Burley*[b]

[a]*GSK, Gunnels Wood Road, Stevenage, Hertfordshire, SG1 2NY, United Kingdom*
[b]*Department of Pure and Applied Chemistry, University of Strathclyde, Glasgow, G1 1XL, United Kingdom*

Table of Contents

Figure S1. .... **2**
Figure S2. .... **2**
Figure S3. .... **3**
Figure S4. .... **3**
Figure S5. .... **3**
Figure S6. .... **4**
Figure S7. .... **5**
Figure S8. .... **6**
Figure S9. .... **7**
1. Chemistry **8**
1.1 General experimental procedures 8
1.2 RIPK2 PROTAC **1** 10
1.3 Dibromopyridazinedione **S3** 11
1.4 Boc-Val-Cit-PAB linker **S6** 14
1.5 Conjugation reagent: diBrPD-VC-PABC-PROTAC **S10** 17
1.6 LCMS Traces 22
1.7 NMR Spectra 24
2. Biology and Bioconjugation **32**
2.1 Materials and methods 32
2.2 Expression of mAbs 33
2.3 Synthesis of ADC-**2** and ADC-**3** 34
3. Biological Assays **35**
3.1 Materials and Methods 35
3.2 Uncropped blots 36
4. References **39**

Supplementary Figures

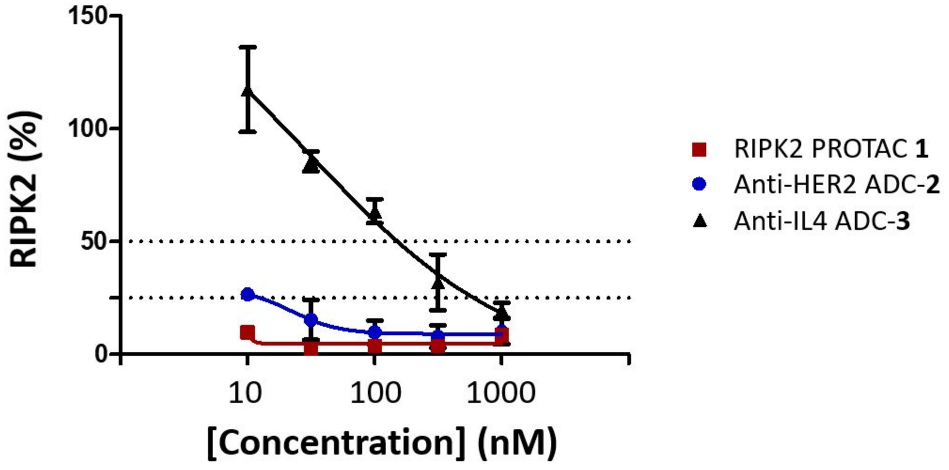

Figure S1. RIPK2 band quantification of Western Blots after a 16 h incubation of SKOV3 cells with PROTAC **1**, ADC-**2** and ADC-**3** (mean ± SD, *n* = 2).

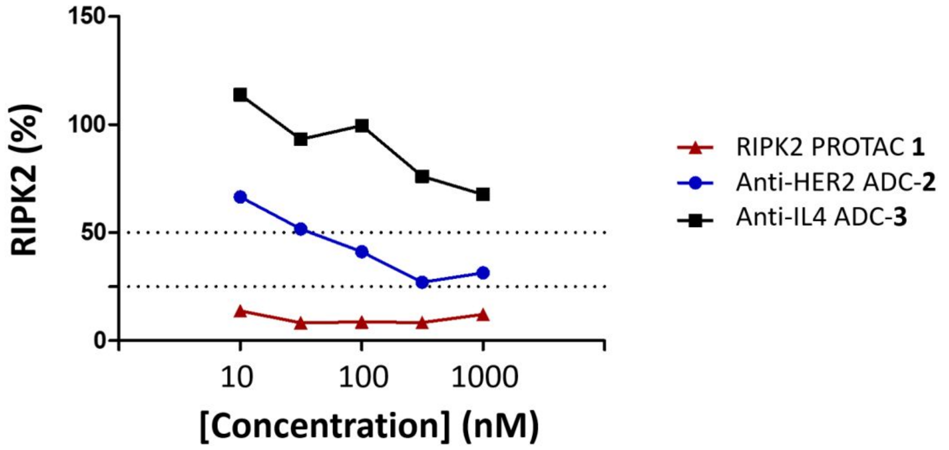

Figure S2. RIPK2 band quantification of Western Blots after a 6 h incubation of SKOV3 cells with PROTAC **1**, ADC-**2** and ADC-**3**.

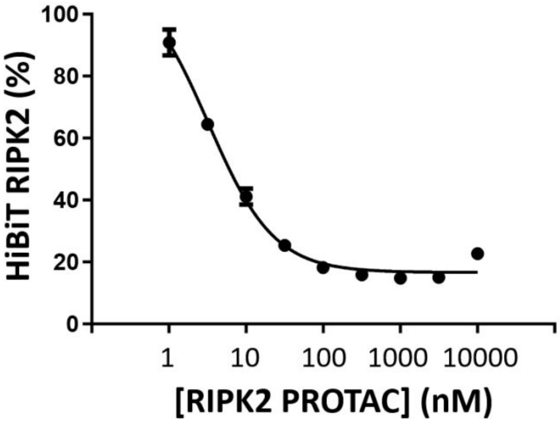

**DC50 (nM)**	6
**Dmax (%)**	83.4
**R squared**	0.9767

Figure S3. RIPK2 levels in a RIPK2 HiBiT HEK293 cell line after a 16 h incubation with PROTAC **1**. RIPK2 levels determined using the Promega Nano-Glo HiBiT Lytic Detection system (mean ± SD, *n* = 3).

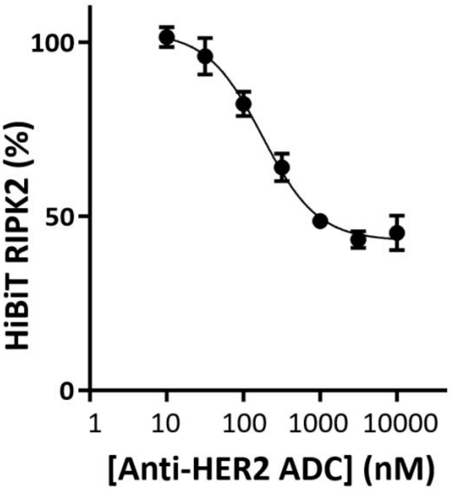

**DC50 (nM)**	955
**Dmax (%)**	57.0
**R squared**	0.9803

Figure S4. RIPK2 levels in a RIPK2 HiBiT HEK293 cell line after a 16 h incubation with ADC-**2**. RIPK2 levels determined using the Promega Nano-Glo HiBiT Lytic Detection system (mean ± SD, *n* = 3).

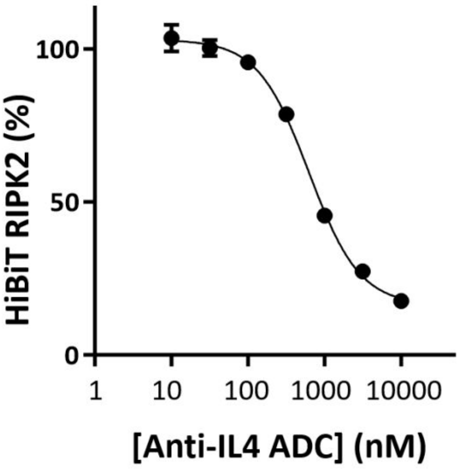

**DC50 (nM)**	887
**Dmax (%)**	83.7
**R squared**	0. 9956

Figure S5. RIPK2 levels in a RIPK2 HiBiT HEK293 cell line after a 16 h incubation with ADC-**3**. RIPK2 levels determined using the Promega Nano-Glo HiBiT Lytic Detection system (mean ± SD, *n* = 3).

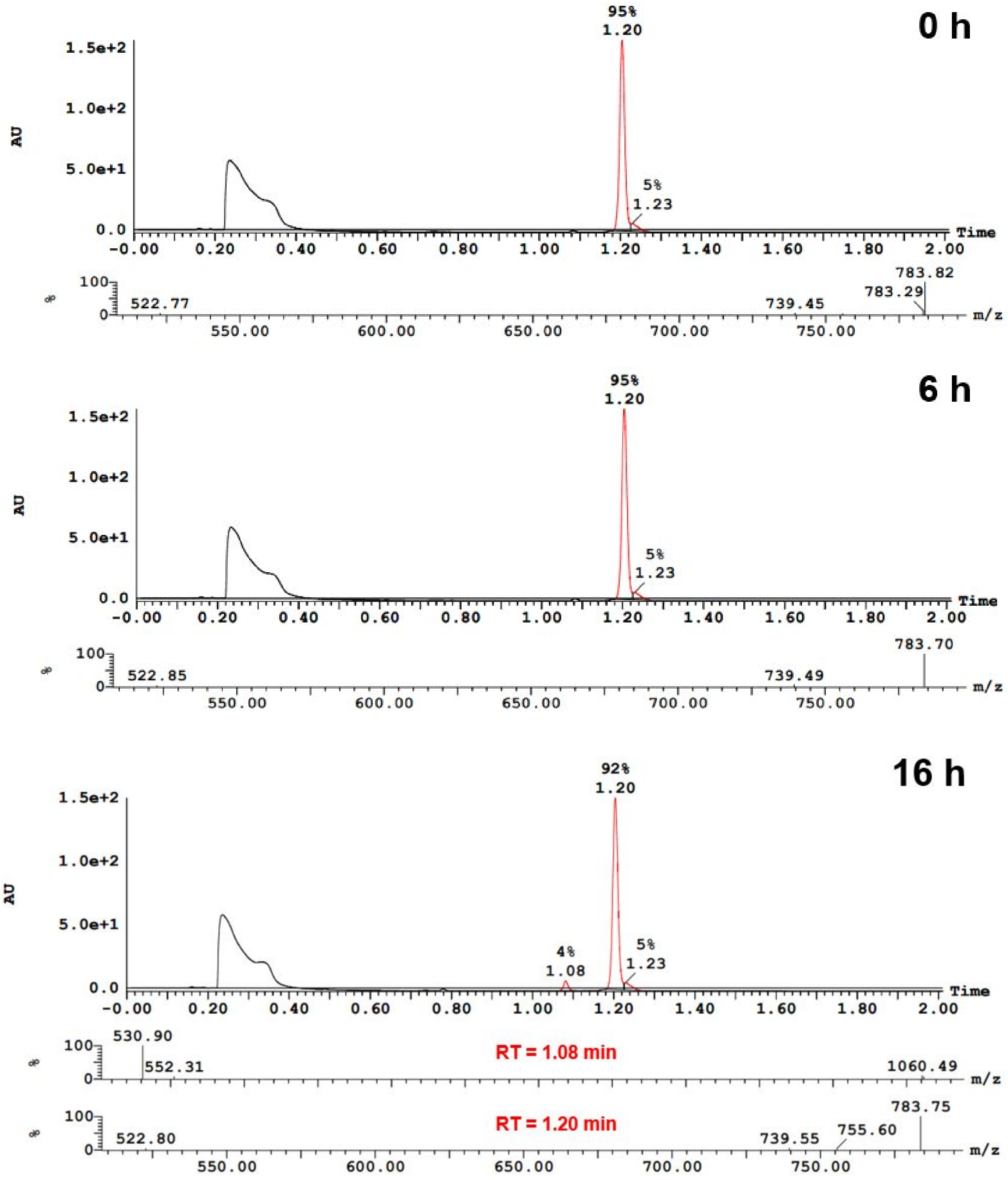

Figure S6. High pH LCMS analysis of **S8** after incubation at 37 °C in 1:1 DMF/McCoy's 5A medium supplemented with glutamine, 10% FBS and 1% Penicillin-Streptomycin. LCMS taken at t = 0, 6 and 16 h time points. Expected $[(M+2H/2]^{+}$ of **S8**: 783.3, expected $[M+H]^{+}$ of PROTAC **1**: 1060.4.

A

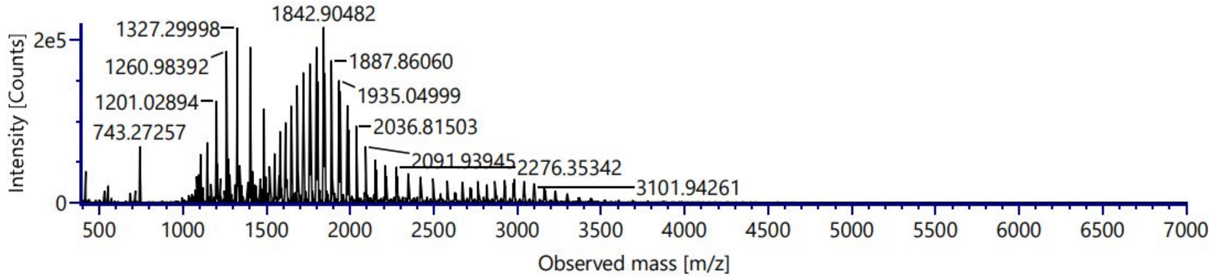

B

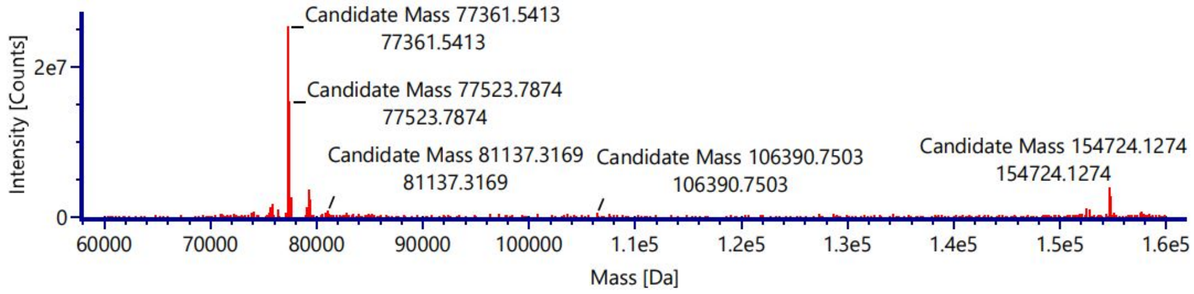

C

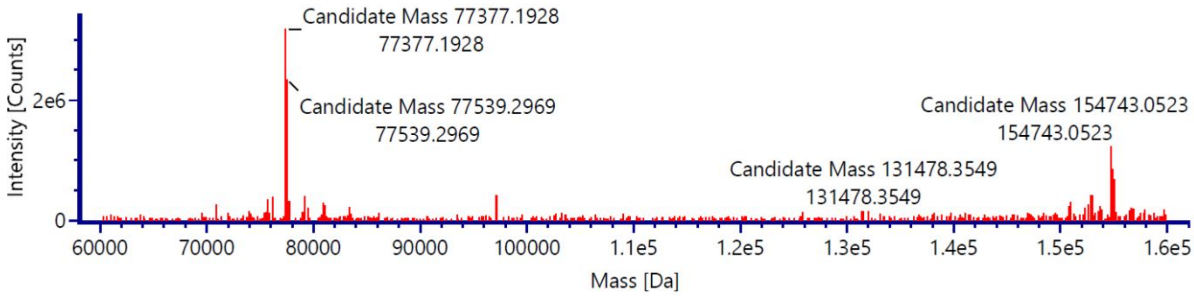

Figure S7. Intact MS of ADC-**2**[1]: **(A)** Non-deconvoluted ion-series, **(B)** deconvoluted MS, **(C)** deconvoluted MS after 275 days stored at 4 °C.

---

[1] The ionisation of the half-mAb is significantly greater than that of the full-mAb due to the tendency for smaller species to ionise more readily. Therefore, the ratio of half-mAb to full-mAb from the mass spec cannot be compared to the corresponding bands in the SDS-PAGE analysis.

A

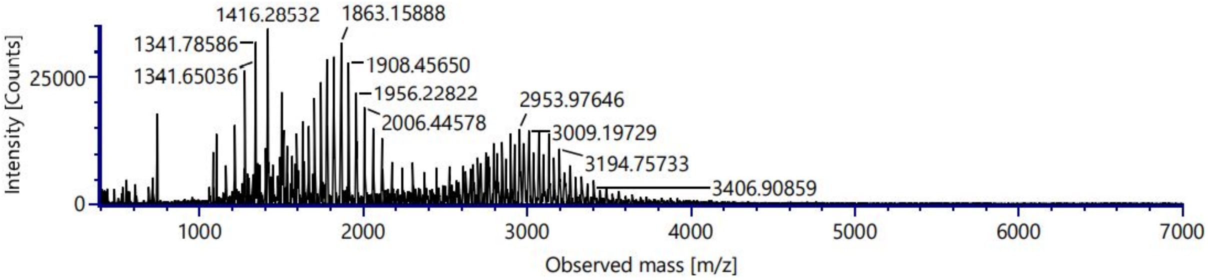

B

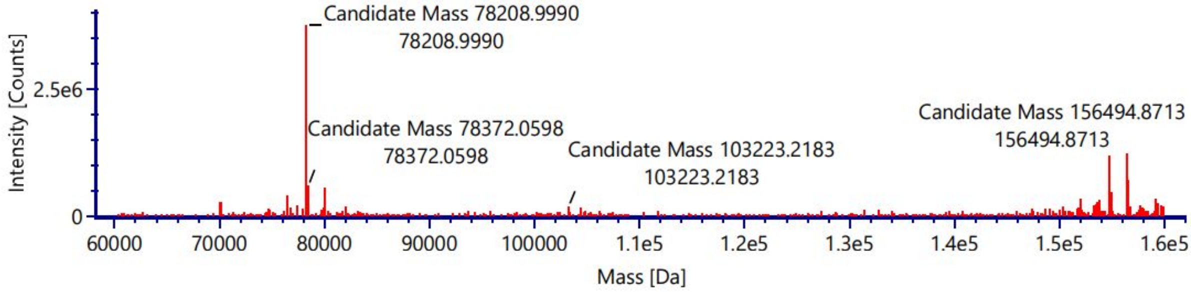

Figure S8. Intact MS of ADC-**3**[2]: **(A)** Non-deconvoluted ion-series, **(B)** deconvoluted MS.

[2] The ionisation of the half-mAb is significantly greater than that of the full-mAb due to the tendency for smaller species to ionise more readily. Therefore, the ratio of half-mAb to full-mAb from the mass spec cannot be compared to the corresponding bands in the SDS-PAGE analysis.

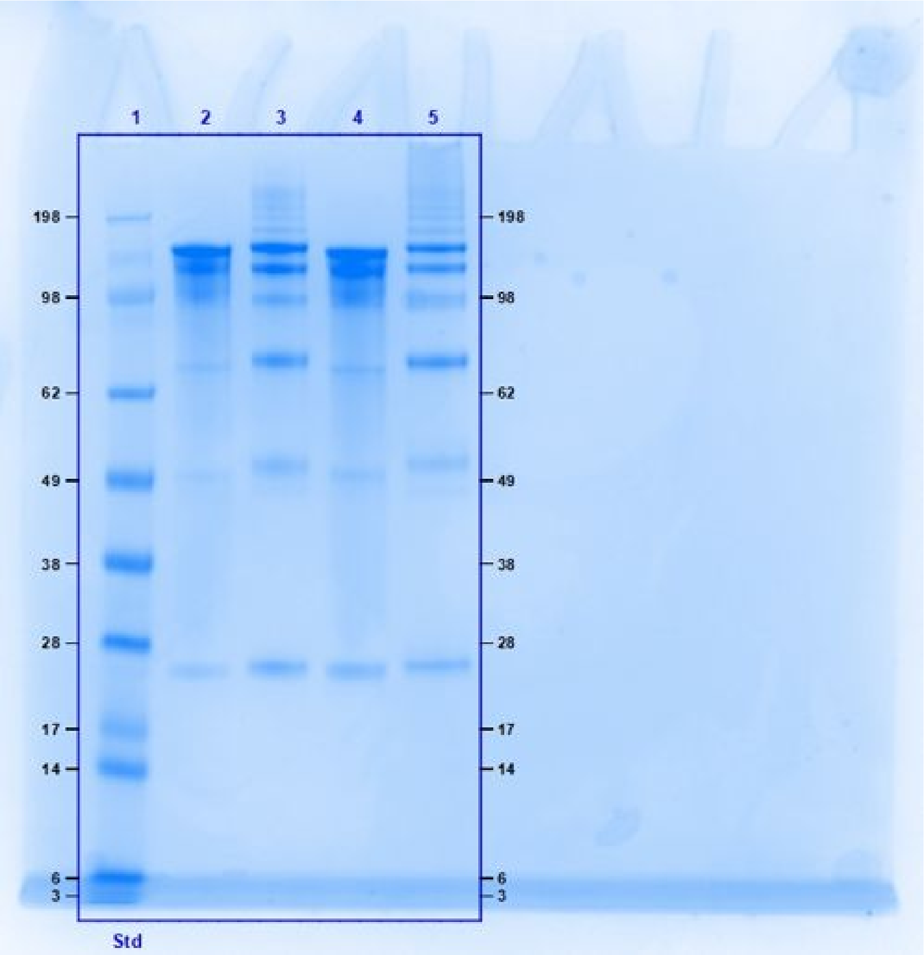

Figure S9. SDS-PAGE of ADC-**2** and ADC-**3**: Lane 1 = Molecular Ladder, Lane 2 = Unmodified anti-IL4 mAb, Lane 3 = ADC-**3**, Lane 4 = Unmodified anti-HER2 mAb, Lane 5 = ADC-**2**.

Aggregation was quantified by densitometry using Image Lab Software 6.1:

ADC-**2**: 17% aggregation

ADC-**3**: 8% aggregation

1. Chemistry

1.1 General experimental procedures

Solvents and reagents

Solvents and reagents were purchased from commercial suppliers and used as received. Reactions were monitored by liquid chromatography-mass spectroscopy (LCMS) or thin-layer chromatography (TLC). Prior to commencing all reactions, reaction vessels were sealed, evacuated, and backfilled with $N_{2(g)}$ (× 3) to ensure the presence of an inert atmosphere.

Thin-layer chromatography (TLC)

TLC was carried out using polyester-backed pre-coated silica plates (0.2 mm particle size). Spots were visualised under ultraviolet light of λ_{max} = 254 nm. In cases where spots were difficult to visualise, the plate was stained with $KMnO_4$ (potassium permanganate) or ninhydrin before gentle heating.

Flash column chromatography

Column chromatography was carried out using the Teledyne ISCO Combi*Flash*® R*f+* apparatus with Redi*Sep*® silica cartridges (normal-phase), Biotage® SNAP KP-C18 cartridges (reverse-phase) or an EZ Prep® column (preparatory HPLC). Eluent conditions are stated in a form describing a gradient of the minor solvent (e.g. EtOAc) in the major solvent (e.g. cyclohexane).

Liquid chromatography mass spectrometry (LCMS)

LCMS analysis was completed on a Waters® Acquity UPLC instrument equipped with a BEH (ethylene-bridged hybrid) column (50 mm × 2.1 mm with 1.7 µm packing diameter) and a Waters® Micromass ZQ MS using alternate-scan positive and negative electrospray ionisation. Analytes were detected as a summed UV wavelength spectra between 210-350 nm. Mass to charge (m/z) ratios are shown in Daltons. Two LCMS methods were used:

- **Formic:** 40 °C, 1 mL/min flow rate, using a mobile phase gradient of water containing 0.1% formic acid (v/v) and acetonitrile containing 0.1% formic acid (v/v). Gradient conditions were initially 1% of the acetonitrile mixture, increasing linearly to 97% over 1.5 min, before remaining at 97% for 0.4 min, then rising to 100% over 0.1 min.
- **High pH:** 40 °C, 1 mL/min flow rate, using a mobile phase gradient of water containing aq. ammonium bicarbonate (10 mM, adjusted to pH 10 with 0.88 M aqueous ammonia) and acetonitrile. Gradient conditions were initially 1% of the acetonitrile mixture, increasing linearly to 97% over 1.5 min, before remaining at 97% for 0.4 min, then rising to 100% over 0.1 min.

High-resolution mass spectrometry (HRMS)

HRMS analysis were conducted on a Waters XEVO G2-XS quadrupole time-of-flight (QTof) mass spectrometer instrument. Mass to charge (m/z) ratios are shown in Daltons. LCMS analysis has been carried out using one of the following methods:

- **10 min Formic:** Ionisation mode: Positive Electrospray. Acquity UPLC CSH C18 column (100 mm x 2.1 mm, 1.7 µm packing diameter) at 50 °C, 0.8 mL/min flow rate. Gradient elution with the eluents as water containing 0.1% volume/volume (v/v) formic acid and (B) MeCN. The UV detection was a summed signal from wavelength of 210 nm to 350 nm. Injection volume: 0.2 µL. The elution conditions began with 5% MeCN mixture, increasing to 93% over 6 minutes, before remaining at 93% for 0.5 minutes, decreasing back to 5% MeCN mixture for 0.5 minutes before equilibrating for 0.5 minutes.
- **20 min High pH:** Ionisation mode: Positive Electrospray. Acquity UPLC BEH C18 column (100 mm x 2.1 mm, 1.7 µm packing diameter) at 50 °C, 0.8 mL/min flow rate. Gradient elution with the eluents as 10 mM ammonium bicarbonate in water adjusted to pH 10 with ammonia solution and MeCN. The UV detection was a summed signal from wavelength of 210 nm to 500 nm. Injection volume: 0.2 µL. The elution conditions began with 1% MeCN mixture, increasing to 90% over 17 minutes, before remaining at 90% for 1.5 minutes, decreasing back to 1% MeCN mixture for 1.0 minutes before equilibrating for 1.0 minutes.

Nuclear magnetic resonance spectroscopy (NMR)

Proton (^{1}H) and carbon (^{13}C) spectra were measured on a Bruker AV400 (^{1}H = 400 MHz, ^{13}C = 101 MHz) spectrometer. Chemical shifts are reported in ppm, relative to the chemical shift of tetramethylsilane (TMS = 0.00 ppm) or the following solvent peaks: $CDCl_3$ (^{1}H = 7.26 ppm, ^{13}C = 77.2 ppm), CD_3OD (^{1}H = 3.31 ppm, ^{13}C = 49.0 ppm), or $(CD_3)_2SO$ (^{1}H = 2.50 ppm, ^{13}C = 39.5 ppm). Peak assignments are stated as chemical shifts, integrations, and coupling constants (where relevant). Coupling constants are quoted to the nearest 0.1 Hz and multiplicities described as either singlet (s), doublet (d), triplet (t), quartet (q), quintet (quin), sextet (sxt), septet (sept), broad (br), or multiplet (m).

1.2 RIPK2 PROTAC **1**

(2*S*,4*R*)-1-((*S*)-17-((4-(Benzo[*d*]thiazol-5-ylamino)-6-(*tert*-butylsulfonyl)quinolin-7-yl)oxy)-2-(*tert*-butyl)-4-oxo-6,9,12,15-tetraoxa-3-azaheptadecanoyl)-4-hydroxy-*N*-(4-(4-methylthiazol-5-yl)benzyl)pyrrolidine-2-carboxamide (1)

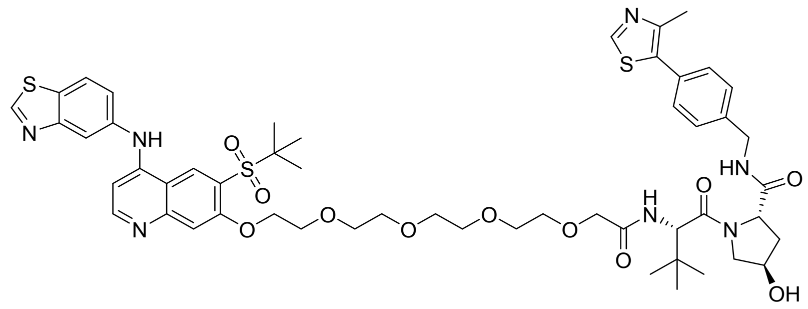

^{1}H NMR (400 MHz, DMSO-d_6): δ 9.50 - 9.41 (m, 1H), 9.02 (s, 1H), 8.99 - 8.93 (m, 1H), 8.58 (br t, *J* = 6.1 Hz, 1H), 8.48 (d, *J* = 5.8 Hz, 1H), 8.24 (d, *J* = 8.7 Hz, 1H), 8.07 (d, *J* = 2.1 Hz, 1H), 7.55 (dd, *J* = 2.1, 8.7 Hz, 1H), 7.48 (s, 1H), 7.44 - 7.35 (m, 5H), 6.87 (d, *J* = 5.8 Hz, 1H), 5.14 (br s, 1H), 4.57 (d, *J* = 9.5 Hz, 1H), 4.49 - 4.31 (m, 3H), 4.30 - 4.22 (m, 1H), 3.97 (s, 2H), 3.87 - 3.81 (m, 2H), 3.72 - 3.48 (m, 16H), 2.44 (s, 3H), 2.11 - 2.03 (m, 1H), 1.96 - 1.87 (m, 1H), 1.40 - 1.30 (m, 9H), 1.00 - 0.90 (m, 9H). Aniline NH not observed.

LCMS (High pH): t_R = 1.08 min, ([M+H]$^+$ 1060.1, (99% purity).

The synthesis of **S1** was carried out as reported in *Nat. Chem. Biol.* **2015,** *11*, 611-617.[1]

1.3 Dibromopyridazinedione **S3**

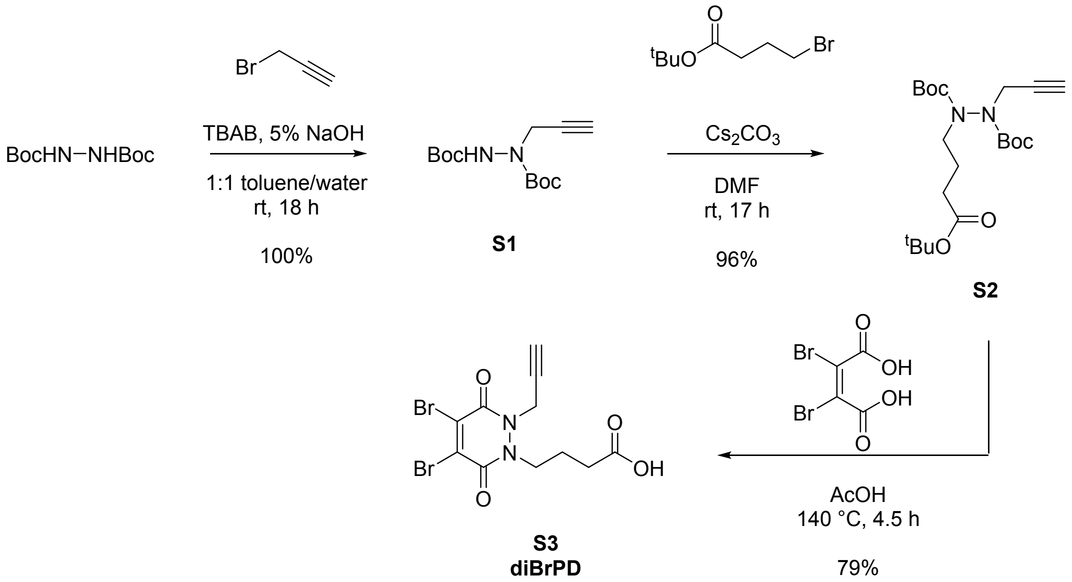

Scheme S1. Synthesis of diBrPD **S3**.

Di-*tert*-butyl 1-(prop-2-yn-1-yl)hydrazine-1,2-dicarboxylate (S1)

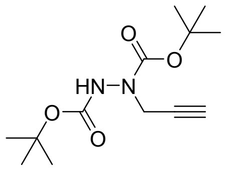

To a suspension of di-tert-butyl hydrazine-1,2-dicarboxylate (3 g, 12.9 mmol) in toluene (20 mL) was added tetrabutylammonium bromide (0.125 g, 0.39 mmol), 3-bromoprop-1-yne (4.32 ml, 38.7 mmol) and 5% aqueous NaOH (20 mL) and the resulting biphasic mixture stirred at rt for 18 h. The mixture was diluted with water (150 mL) and extracted with ethyl acetate (3 × 80 mL). The combined organics were washed with brine (150 mL), passed through a hydrophobic frit and concentrated *in vacuo*. The residue was dried under vacuum to afford **S1** as an orange solid (3.49 g, 12.9 mmol, 100% yield).

^{1}H NMR (400 MHz, $CDCl_3$): δ 6.51 (br s, 1H), 4.28 (br s, 2H), 2.25 (t, *J* = 2.6 Hz, 1H), 1.57 - 1.43 (m, 18H) (Major rotamer reported).

^{13}C NMR (101 MHz, $CDCl_3$): δ 154.6, 81.9, 81.5, 78.7, 72.0, 39.4, 28.2, 28.1.

IR ν_{max} (neat): 3310, 3291, 2980, 2937, 1728, 1689, 1513 cm^{-1}.

m.p.: 98-100 °C.

TLC (1:1 EtOAc/cyclohexane, visualisation = $KMnO_4$)**:** R_f = 0.52.

Di-*tert*-butyl 1-(4-(*tert*-butoxy)-4-oxobutyl)-2-(prop-2-yn-1-yl)hydrazine-1,2-dicarboxylate (S2)

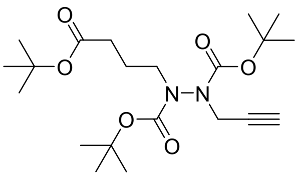

To a stirred solution of **S1** (3.4 g, 12.6 mmol) in DMF (50 mL) was added Cs_2CO_3 (6.15 g, 18.9 mmol) and tert-butyl 4-bromobutanoate (2.5 mL, 13.2 mmol). The resulting mixture was stirred at rt for 17 h. The mixture was diluted with water (200 mL) and extracted with ethyl acetate (3 × 80 mL). The combined organics were washed with 5% aqueous LiCl (3 × 50 mL), passed through a hydrophobic frit and concentrated *in vacuo*. The oil was purified by flash column chromatography, eluting 0-15% EtOAc in cyclohexane on a 80 g silica column over 20 CV. Fractions containing product were combined and concentrated *in vacuo* to afford **S2** as a light yellow oil (4.99 g, 12.1 mmol, 96% yield).

^{1}H NMR (400 MHz, $CDCl_3$): δ 4.69 - 4.28 (m, 1H), 4.25 - 3.92 (m, 1H), 3.68 - 3.30 (m, 2H), 2.38 - 2.18 (m, 3H), 2.04 - 1.85 (m, 2H), 1.74 - 1.24 (m, 27H). Rotamers observed.

^{13}C NMR (101 MHz, $CDCl_3$): δ 172.4, 154.6, 154.4, 81.8, 81.1, 80.1, 78.4, 72.8, 49.3, 39.3, 33.2, 28.24, 28.19, 28.1, 23.4. Rotamers observed.

HRMS (ESI): calculated for $C_{21}H_{36}N_2O_6Na$ (*m/z*) $[M+Na]^+$ requires 435.2471, found $[M+Na]^+$ 435.2471 (error 0.0 ppm).

IR ν_{max} (neat): 3262, 2977, 2933, 1709, 1478, 1456 cm^{-1}.

TLC (3:7 EtOAc/Cyclohexane, visualisation = $KMnO_4$)**:** R_f = 0.46.

4-(4,5-Dibromo-3,6-dioxo-2-(prop-2-yn-1-yl)-3,6-dihydropyridazin-1(2*H*)-yl)butanoic acid (S3)

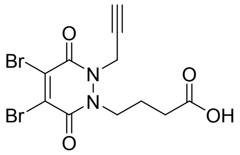

A solution of 2,3-dibromomaleic acid (1 g, 3.65 mmol) in AcOH (25 mL) was heated to reflux and left to stir for 30 min prior to addition of **S2** (1.3 g, 3.15 mmol) in AcOH (5 mL). The solution turned from colourless to brown. The reaction mixture was heated to reflux for an additional 4 h before concentration *in vacuo* to give a brown oil. The crude product was purified by flash column

chromatography, eluting 25-60% EtOAc [1% AcOH] in cyclohexane on a 120 g silica column over 20 CV. Fractions containing product were combined and concentrated *in vacuo* to afford **S3** as a pale yellow solid (985 mg, 2.5 mmol, 79% yield).

^{1}H NMR (400 MHz, CD_3OD): δ 5.06 (d, *J* = 2.4 Hz, 2H), 4.34 - 4.25 (m, 2H), 2.95 (t, *J* = 2.4 Hz, 1H), 2.44 (t, *J* = 6.8 Hz, 2H), 2.07 - 1.97 (m, 2H). OH not observed.

^{13}C NMR (101 MHz, CD_3OD): δ 174.7, 153.6, 153.2, 136.1, 134.9, 75.8, 74.5, 46.9, 36.8, 29.9, 22.5.

LCMS (Formic): t_R = 0.72 min, $[M(Br^{79}Br^{79})+H]^+$ 393.1, $[M(Br^{79}Br^{81})+H]^+$ 395.0, $[M(Br^{81}Br^{81})+H]^+$ 397.0, (100% purity).

HRMS (ESI): calculated for $C_{11}H_{11}Br_2N_2O_4$ (*m/z*) $[M+H]^+$ requires 392.9086, found $[M+H]^+$ 392.9085 (error -0.3 ppm).

IR v_{max} (neat): 3287, 2938, 2131, 1693, 1639, 1575 cm^{-1}.

1.4 Boc-Val-Cit-PAB linker **S6**

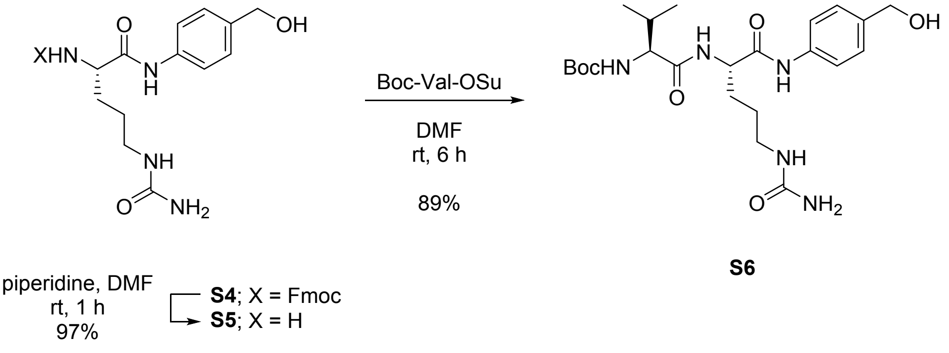

Scheme S2. Synthesis of Boc-VC-PAB linker **S6**.

(9*H*-Fluoren-9-yl)methyl (*S*)-(1-((4-(hydroxymethyl)phenyl)amino)-1-oxo-5-ureidopentan-2-yl)carbamate (S4)

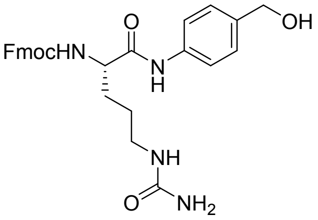

^{1}H NMR (400 MHz, DMSO-d_6): δ 10.08 - 9.91 (m, 1H), 7.90 (d, *J* = 7.6 Hz, 2H), 7.80 - 7.71 (m, 2H), 7.64 (d, *J* = 8.1 Hz, 1H), 7.57 (d, *J* = 8.6 Hz, 2H), 7.46 - 7.39 (m, 2H), 7.38 - 7.30 (m, 2H), 7.25 (d, *J* = 8.6 Hz, 2H), 5.98 (br t, *J* = 5.6 Hz, 1H), 5.41 (s, 2H), 5.08 (t, *J* = 5.6 Hz, 1H), 4.44 (d, *J* = 5.6 Hz, 2H), 4.33 - 4.12 (m, 4H), 3.12 - 2.90 (m, 2H), 1.76 - 1.56 (m, 2H), 1.55 - 1.34 (m, 2H).

LCMS (High pH): t_R = 0.96 min, $[M+H]^+$ 503.1, (98% purity).

The synthesis of **S4** was carried out as reported in *Angew. Chem., Int. Ed. Engl.* **2021,** *60*, 21691-21696.[2]

(*S*)-2-Amino-*N*-(4-(hydroxymethyl)phenyl)-5-ureidopentanamide (S5)

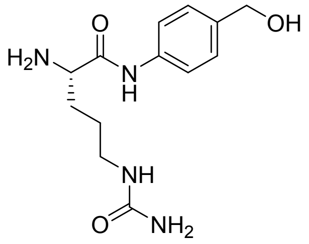

To a solution of **S4** (6.22 g, 12.4 mmol) in DMF (20 mL) was added piperidine (2.45 ml, 24.8 mmol), and the resulting solution stirred at rt for 1 h. The reaction mixture was diluted with water (100 mL) and filtered under vacuum, washing the solid with additional water (100 mL). The filtrate was washed with diethyl ether (50 mL), then EtOAc (50 mL), and the aqueous layer was then concentrated *in vacuo* to afford **S5** as a white solid (3.45 g, 11.9 mmol, 97% yield).

^{1}H NMR (400 MHz, DMSO-*d*$_6$): δ 10.37 - 9.42 (m, 1H), 7.58 (d, *J* = 8.6 Hz, 2H), 7.24 (d, *J* = 8.6 Hz, 2H), 5.94 (br t, *J* = 5.6 Hz, 1H), 5.35 (s, 2H), 5.17 - 4.97 (m, 1H), 4.44 (s, 2H), 3.31 - 3.27 (m, 1H), 3.05 - 2.91 (m, 2H), 1.70 - 1.56 (m, 1H), 1.55 - 1.34 (m, 3H). NH_2 protons not observed.

^{13}C NMR (101 MHz, DMSO-*d*$_6$): δ 174.8, 159.2, 138.0, 137.7, 127.4, 119.3, 63.1, 55.7, 33.1, 27.2.

LCMS (High pH): t_R = 0.40 min, $[M+H]^+$ 281.3, (100% purity).

HRMS (ESI): calculated for $C_{13}H_{21}N_4O_3$ (*m/z*) $[M+H]^+$ requires 281.1614, found $[M+H]^+$ 281.1602 (error -4.3 ppm).

IR ν_{max} (neat): 3304, 2929, 2865, 1654, 1605, 1541 cm^{-1}.

***Tert*-butyl ((*S*)-1-(((*S*)-1-((4-(hydroxymethyl)phenyl)amino)-1-oxo-5-ureidopentan-2-yl)amino)-3-methyl-1-oxobutan-2-yl)carbamate (S6)**

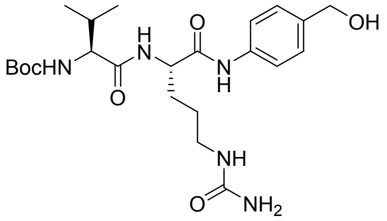

S5 (260 mg, 0.93 mmol) and Boc-Val-OSu (292 mg, 0.93 mmol) were combined in DMF (1 mL) and the resulting mixture stirred at rt for 6 h. The reaction mixture was purified directly by reverse phase chromatography, eluting 15-55% acetonitrile in water with a 10 mM ammonium bicarbonate modifier

adjusted to pH 10. Fractions containing product were combined and concentrated *in vacuo* to afford **S6** as an off-white solid (394 mg, 0.82 mmol, 89% yield).

^{1}H NMR (400 MHz, DMSO-d_6): δ 9.96 (s, 1H), 7.95 (br d, J = 7.8 Hz, 1H), 7.54 (d, J = 8.6 Hz, 2H), 7.24 (d, J = 8.6 Hz, 2H), 6.74 (br d, J = 8.8 Hz, 1H), 5.96 (t, J = 5.9 Hz, 1H), 5.39 (s, 2H), 5.07 (t, J = 5.6 Hz, 1H), 4.44 (d, J = 5.6 Hz, 3H), 3.84 (br t, J = 7.7 Hz, 1H), 3.10 - 2.99 (m, 1H), 2.99 - 2.90 (m, 1H), 2.03 - 1.92 (m, 1H), 1.77 - 1.66 (m, 1H), 1.65 - 1.53 (m, 1H), 1.51 - 1.31 (m, 11H), 0.87 (d, J = 6.8 Hz, 3H), 0.83 (d, J = 6.6 Hz, 3H).

^{13}C NMR (101 MHz, DMSO-d_6): δ 171.8, 170.8, 159.3, 156.0, 137.95, 137.92, 127.4, 119.4, 78.6, 63.1, 60.2, 53.4, 39.1, 30.9, 30.2, 28.7, 27.2, 19.7, 18.6.

LCMS (High pH): t_R = 0.75 min, $[M+H]^+$ 480.3, (100% purity).

HRMS (ESI): calculated for $C_{23}H_{38}N_5O_6$ (*m/z*) $[M+H]^+$ requires 480.2822, found 480.2820 (error -0.4 ppm).

IR ν_{max} (neat): 3453, 3301 2965, 2915, 2871, 1692, 1634, 1600, 1527, 1449 cm^{-1}.

1.5 Conjugation reagent: diBrPD-VC-PABC-PROTAC **S10**

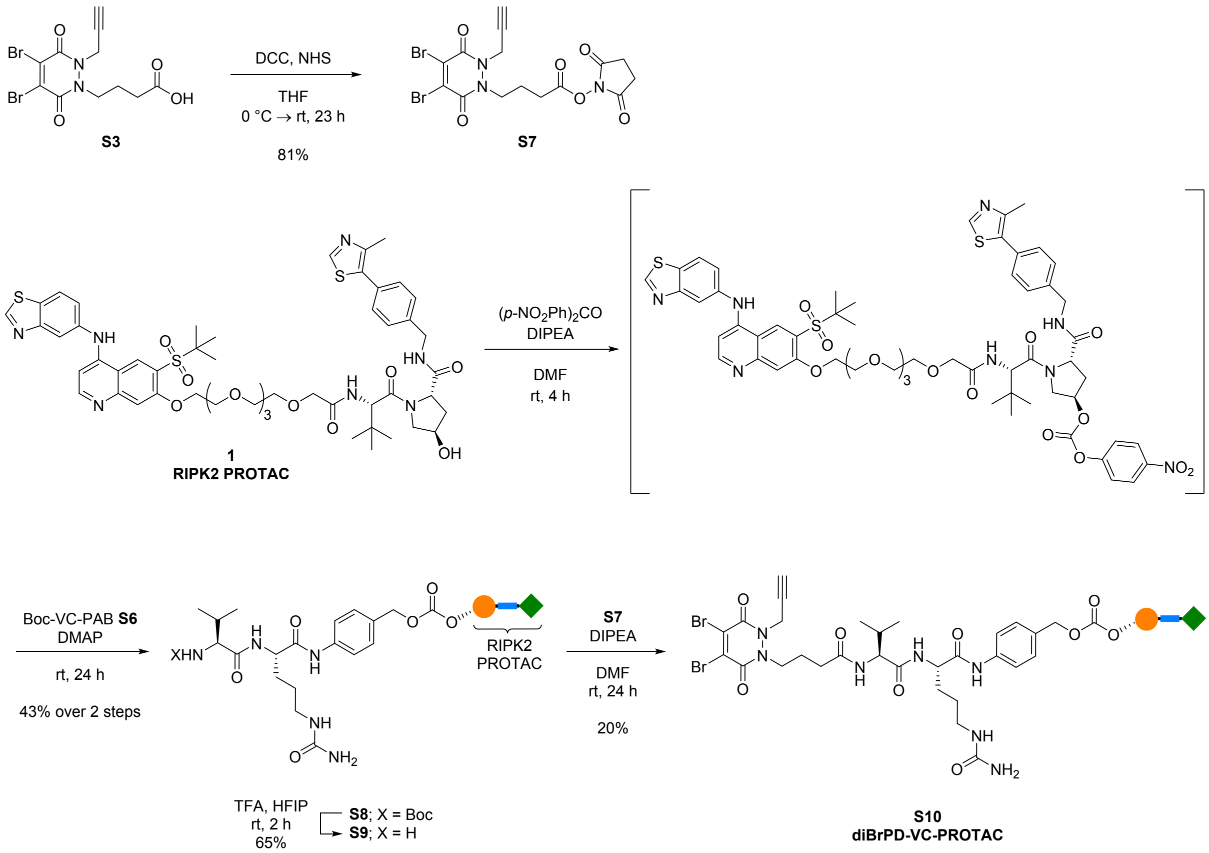

Scheme S3. Synthesis of conjugation reagent diBrPD-VC-PABC-PROTAC **S10**.

2,5-Dioxopyrrolidin-1-yl 4-(4,5-dibromo-3,6-dioxo-2-(prop-2-yn-1-yl)-3,6-dihydropyridazin-1(2*H*)-yl)butanoate (S7)

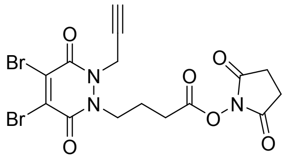

A solution of **S3** (269 mg, 0.68 mmol) in THF (8.5 mL) was cooled to 0 °C and to this was added DCC (155 mg, 0.75 mmol). The mixture was stirred at 0 °C for 30 min, then *N*-hydroxysuccinimide (86 mg, 0.75 mmol) was added and the resulting mixture stirred at rt for 23 h. The suspension was filtered and the filtrate concentrated *in vacuo*. The residue was purified by column chromatography, eluting 20-80% EtOAc in cyclohexane on a 24 g silica column. Fractions containing product were combined and concentrated *in vacuo* to afford **S7** as a white solid (271 mg, 0.55 mmol, 81% yield).

^{1}H NMR (400 MHz, $CDCl_3$): δ 4.94 (d, *J* = 2.5 Hz, 2H), 4.33 - 4.22 (m, 2H), 2.84 (s, 4H), 2.76 (t, *J* = 6.9 Hz, 2H), 2.42 (t, *J* = 2.5 Hz, 1H), 2.20 - 2.11 (m, 2H).

^{13}C NMR (101 MHz, $CDCl_3$): δ 168.8, 167.9, 153.5, 153.1, 136.8, 135.5, 75.6, 75.0, 46.5, 37.2, 28.0, 25.6, 22.7.

LCMS (Formic): t_R = 0.82 min, $[M(Br^{79}Br^{79})+H]^+$ 489.8, $[M(Br^{79}Br^{81})+H]^+$ 491.8, $[M(Br^{81}Br^{81})+H]^+$ 493.8, (98% purity).

HRMS (ESI): calculated for $C_{15}H_{14}Br_2N_3O_6$ (*m/z*) $[M+H]^+$ requires 489.9249, found $[M+H]^+$ 489.9263 (error 2.4 ppm).

IR v_{max} (neat): 3261, 2946, 1812, 1780, 1731, 1634, 1575 cm^{-1}.

***Tert*-butyl((*S*)-1-(((*S*)-1-((4-((((((3*R*,5*S*)-1-((*S*)-17-((4-(benzo[*d*]thiazol-5-ylamino)-6-(*tert*-butylsulfonyl)quinolin-7-yl)oxy)-2-(*tert*-butyl)-4-oxo-6,9,12,15-tetraoxa-3-azaheptadecanoyl)-5-((4-(4-methylthiazol-5-yl)benzyl)carbamoyl)pyrrolidin-3-yl)oxy)carbonyl)oxy)methyl)phenyl)amino)-1-oxo-5-ureidopentan-2-yl)amino)-3-methyl-1-oxobutan-2-yl)carbamate (S8)**

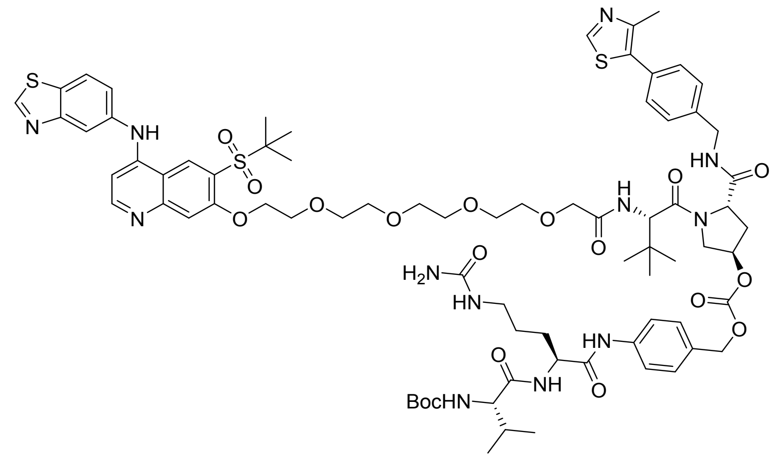

To a solution of **1** (100 mg, 0.094 mmol) in DMF (1 mL) was added bis(4-nitrophenyl)carbonate (72 mg, 0.24 mmol) and DIPEA (0.028 mL, 0.16 mmol) and the resulting mixture stirred at rt for 4 h. **S6** (68 mg, 0.14 mmol) and DMAP (12 mg, 0.094 mmol) was added and the resulting solution stirred at rt for 24 h. The solution was purified by reverse phase chromatography on an XSelect CSH Prep C18 5 µm OBD column, eluting 30-85% acetonitrile in water with a 10 mM ammonium bicarbonate modifier adjusted to pH 10. Fractions containing product were combined and concentrated *in vacuo* to afford **S8** as a yellow amorphous solid (63 mg, 0.04 mmol, 43% yield).

^{1}H NMR (400 MHz, DMSO-d_6): δ 10.09 (s, 1H), 9.67 (s, 1H), 9.43 (s, 1H), 8.97 (s, 1H), 8.95 (s, 1H), 8.62 (br t, *J* = 5.9 Hz, 1H), 8.50 (d, *J* = 5.4 Hz, 1H), 8.20 (d, *J* = 8.6 Hz, 1H), 8.04 (d, *J* = 2.0 Hz, 1H), 7.99 (br d, *J* = 7.6 Hz, 1H), 7.61 (d, *J* = 8.6 Hz, 2H), 7.54 (dd, *J* = 8.6, 2.0 Hz, 1H), 7.48 (s, 1H), 7.45 - 7.37 (m, 5H), 7.33 (d, *J* = 8.8 Hz, 2H), 6.90 (d, *J* = 5.6 Hz, 1H), 6.72 (br d, *J* = 8.8 Hz, 1H), 5.96 (br t, *J* = 5.8 Hz, 1H), 5.39

(s, 2H), 5.24 (br s, 1H), 5.10 (d, J = 12.5 Hz, 1H), 5.08 (d, J = 12.0 Hz, 1H), 4.51 - 4.22 (m, 8H), 4.04 (br d, J = 12.2 Hz, 1H), 3.95 (s, 2H), 3.90 - 3.78 (m, 3H), 3.63 - 3.50 (m, 12H), 3.09 - 2.99 (m, 1H), 2.99 - 2.90 (m, 1H), 2.44 (s, 3H), 2.37 - 2.29 (m, 1H), 2.20 - 2.10 (m, 1H), 2.01 - 1.91 (m, 1H), 1.75 - 1.65 (m, 1H), 1.65 - 1.54 (m, 1H), 1.47 - 1.35 (m, 11H), 1.33 (s, 9H), 0.96 (s, 9H), 0.86 (d, J = 6.9 Hz, 3H), 0.82 (d, J = 6.9 Hz, 3H).

^{13}C NMR (151 MHz, DMSO-d_6): δ 171.8, 171.4, 171.1, 169.7, 169.4, 159.3, 157.8, 156.8, 155.9, 154.6, 154.5, 154.2, 153.5, 151.9, 149.9, 148.2, 139.7, 139.5, 139.2, 131.6, 131.2, 130.4, 130.2, 129.7, 129.6, 129.2, 127.9, 123.6, 123.5, 122.2, 119.5, 117.2, 113.8, 110.8, 101.8, 78.6, 77.4, 70.9, 70.4, 70.31, 70.27, 70.1, 70.0, 69.5, 69.0, 68.8, 61.2, 60.2, 58.7, 56.5, 54.0, 53.4, 42.2, 39.1, 35.6, 35.2, 30.9, 30.0, 28.6, 27.2, 26.6, 24.2, 19.7, 18.6, 16.4.

LCMS (High pH): t_R = 1.21 min, ([M+2H]/2)$^+$ 783.6, (100% purity).

HRMS (ESI): calculated for $C_{76}H_{102}N_{12}O_{18}S_3$ (*m/z*) ([M+2H]/2)$^+$ requires 783.3299, found ([M+2H]/2)$^+$ 783.3311 (error 1.5 ppm).

IR ν_{max} (neat): 3278, 2932, 1728, 1635, 1572, 1522 cm^{-1}.

[α_D]$^{20.0\,°C}_{589\,nm}$ (*c* 1.00, MeOH): - 16°

4-((*S*)-2-((*S*)-2-Amino-3-methylbutanamido)-5-ureidopentanamido)benzyl ((3*R*,5*S*)-1-((*S*)-17-((4-(benzo[*d*]thiazol-5-ylamino)-6-(*tert*-butylsulfonyl)quinolin-7-yl)oxy)-2-(*tert*-butyl)-4-oxo-6,9,12,15-tetraoxa-3-azaheptadecanoyl)-5-((4-(4-methylthiazol-5-yl)benzyl)carbamoyl)pyrrolidin-3-yl) carbonate, trifluoroacetic acid salt (S9)

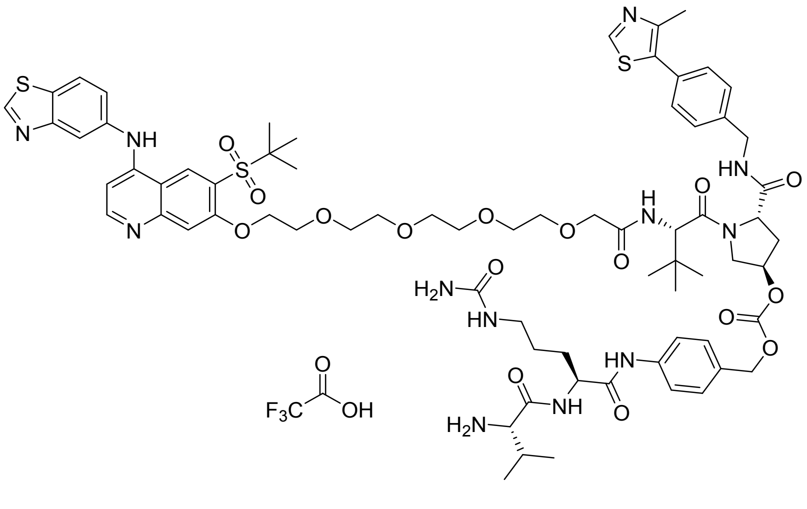

To a solution of **S8** (97 mg, 0.062 mmol) in HFIP (1 mL) was added TFA (48 µL, 0.62 mmol) and the resulting solution stirred at rt for 2 h. The reaction mixture was concentrated under a stream of N_2 to

afford **S9** as a yellow gum (98 mg, 0.04 mmol, 65% yield). The crude product was carried forward with no additional purification.

LCMS (Formic) t_R = 0.72 min, ([M+2H]/2)$^+$ 733.6, (67% purity).

(3*R*,5*S*)-1-((*S*)-17-((4-(Benzo[*d*]thiazol-5-ylamino)-6-(*tert*-butylsulfonyl)quinolin-7-yl)oxy)-2-(*tert*-butyl)-4-oxo-6,9,12,15-tetraoxa-3-azaheptadecanoyl)-5-((4-(4-methylthiazol-5-yl)benzyl)carbamoyl)pyrrolidin-3-yl(4-((*S*)-2-((*S*)-2-(4-(4,5-dibromo-3,6-dioxo-2-(prop-2-yn-1-yl)-3,6-dihydropyridazin-1(2*H*)-yl)butanamido)-3-methylbutanamido)-5-ureidopentanamido)benzyl) carbonate (S10)

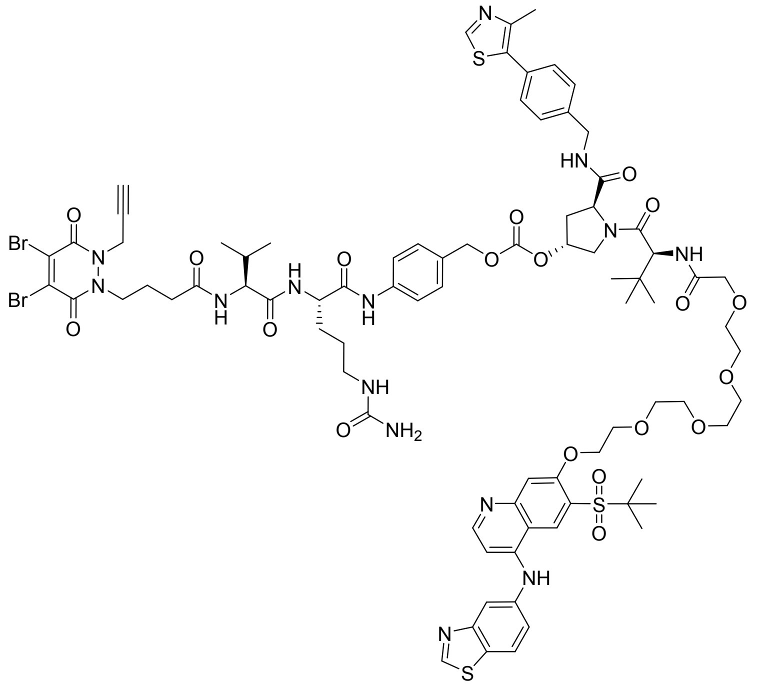

S7 (23 mg, 0.046 mmol) was added to a solution of **S9** (98 mg, 0.042 mmol) and DIPEA (11 µL, 0.062 mmol) in DMF (500 µL), and the resulting solution stirred at rt for 15 h. Additional DIPEA (11 µL, 0.062 mmol) was added and the resulting solution stirred for another 9 h. The solution was purified directly by reverse phase chromatography on an XSelect CSH Prep C18 5 µm OBD column, eluting 30-50% acetonitrile in water with a 10 mM ammonium bicarbonate modifier adjusted to pH 10. Fractions containing product were combined and concentrated under a stream of N_2 to afford **S10** as a yellow solid (15 mg, 8.14 µmol, 20% yield).

^{1}H NMR (600 MHz, DMSO-d_6): δ 9.98 (s, 1 H), 9.69 (br s, 1 H), 9.43 (s, 1 H), 8.97 (s, 1 H), 8.95 (s, 1 H), 8.62 (t, *J* = 6.1 Hz, 1 H), 8.50 (d, *J* = 5.5 Hz, 1 H), 8.20 (d, *J* = 8.4 Hz, 1 H), 8.13 (d, *J* = 7.3 Hz, 1 H), 8.04 (d, *J* = 1.1 Hz, 1 H), 7.91 (d, *J* = 8.4 Hz, 1 H), 7.62 (d, *J* = 8.8 Hz, 2 H), 7.54 (dd, *J* = 8.6, 1.3 Hz, 1 H), 7.48

(s, 1 H), 7.44 - 7.38 (m, 5 H), 7.33 (d, *J* = 8.8 Hz, 2 H), 6.90 (d, *J* = 5.4 Hz, 1 H), 5.96 (br t, *J* = 5.9 Hz, 1 H), 5.39 (s, 2 H), 5.24 (br s, 1 H), 5.10 (d, *J* = 12.2 Hz, 1 H), 5.07 (d, *J* = 12.1 Hz, 1 H), 4.96 (dd, *J* = 18.3, 2.6 Hz, 1 H), 4.92 (dd, *J* = 18.3, 2.6 Hz, 1 H), 4.49 - 4.40 (m, 3 H), 4.40 - 4.36 (m, 1 H), 4.32 (br t, *J* = 4.0 Hz, 2 H), 4.27 (dd, *J* = 15.8, 5.5 Hz, 1 H), 4.22 (dd, *J* = 8.3, 6.8 Hz, 1 H), 4.13 - 4.06 (m, 2 H), 4.04 (br d, *J* = 12.1 Hz, 1 H), 3.95 (s, 2 H), 3.86 (br dd, *J* = 11.9, 3.9 Hz, 1 H), 3.84 - 3.81 (m, 2 H), 3.63 - 3.51 (m, 12 H), 3.46 (t, *J* = 2.4 Hz, 1 H), 3.06 - 2.99 (m, 1 H), 2.99 - 2.92 (m, 1 H), 2.44 (s, 3 H), 2.33 (m, 1 H), 2.31 - 2.21 (m, 2 H), 2.15 (ddd, *J* = 13.9, 9.2, 4.8 Hz, 1 H), 1.98 (dspt, *J* = 6.9, 6.7 Hz, 1 H), 1.85 (quin, *J* = 7.3 Hz, 2 H), 1.75 - 1.68 (m, 1 H), 1.65 - 1.57 (m, 1 H), 1.50 - 1.42 (m, 1 H), 1.41 - 1.35 (m, 1 H), 1.33 (s, 9 H), 0.96 (s, 9 H), 0.88 (d, *J* = 6.6 Hz, 3 H), 0.85 (d, *J* = 6.6 Hz, 3 H).

^{13}C NMR (150 MHz, DMSO-d_6): δ 171.5, 171.1, 170.9, 170.6, 169.2, 168.9, 158.8, 157.3, 154.1, 153.9, 153.7, 153.1, 153.0, 152.9, 152.4, 151.3, 149.4, 147.7, 139.2, 139.1, 138.7, 136.2, 136.1, 134.8, 131.0, 130.7, 129.8, 129.7, 129.2, 129.1, 128.6, 127.4, 123.0, 121.6, 118.9, 116.7, 113.2, 110.2, 101.2, 77.0, 76.8, 76.4, 70.4, 69.82, 69.79, 69.7, 69.5, 69.4, 68.9, 68.5, 68.3, 60.7, 58.2, 57.7, 56.0, 53.5, 53.1, 46.7, 41.7, 38.6, 37.0, 35.1, 34.7, 31.4, 30.4, 29.1, 26.8, 26.0, 23.6, 23.2, 19.1, 18.1, 15.9.

^{15}N NMR: δ 333, 314, 160, 155, 129, 117, 119, 113, 112, 94, 82, 73. Pyrrolidine and quinoline N's not observed.

LCMS (High pH): t_R = 1.17 min, $([M(^{79}Br^{79}Br)+2H]/2)^+$ 920.8, $([M(^{79}Br^{81}Br)+2H]/2)^+$ 921.4, $([M(^{81}Br^{81}Br)+2H]/2)^+$ 922.0, (93% purity).

HRMS (ESI): calculated for $C_{82}H_{101}Br_2N_{14}O_{19}S_3$ (*m/z*) $[M+H]^+$ requires 1839.4898, found 1839.4243 (error 0.5 ppm).

IR ν_{max} (neat): 3263, 2947, 1780, 1728, 1632, 1574, 1404 cm^{-1}.

$[\alpha_D]^{20.0\,°C}_{589\,nm}$ (*c* 0.5, DMSO): - 16°

1.6 LCMS Traces

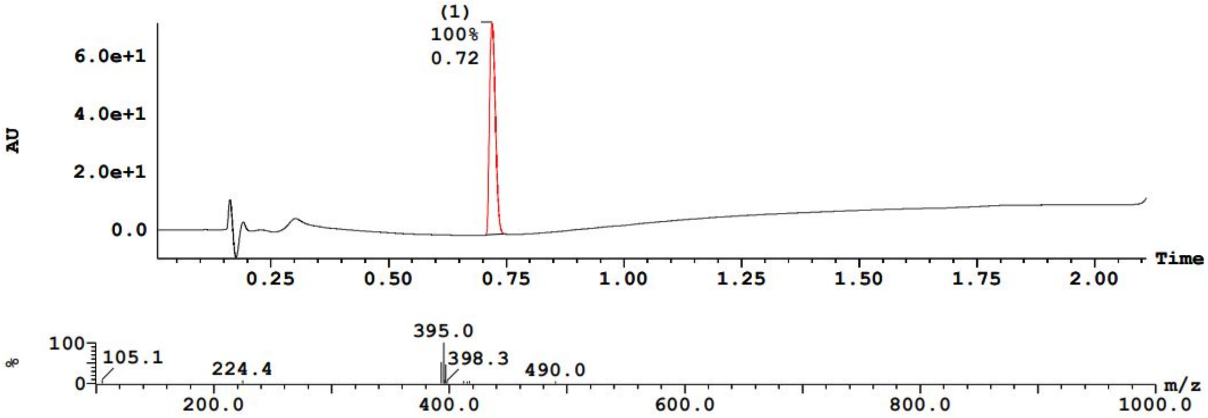

LCMS of S**3.**

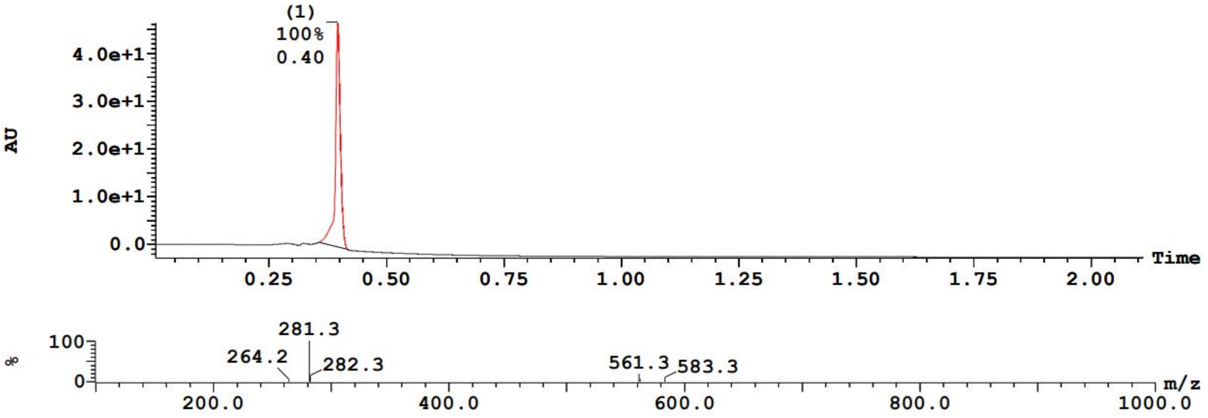

LCMS of S**5**.

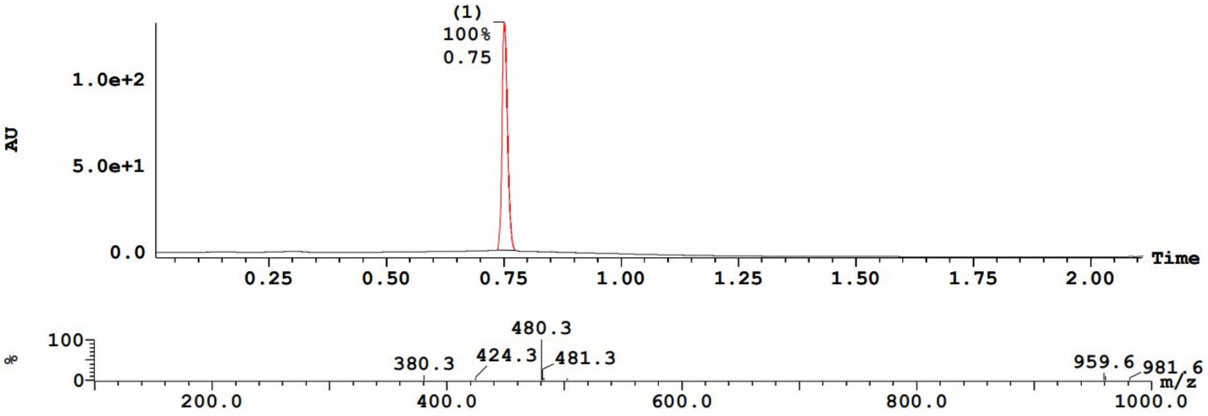

LCMS of S**6**.

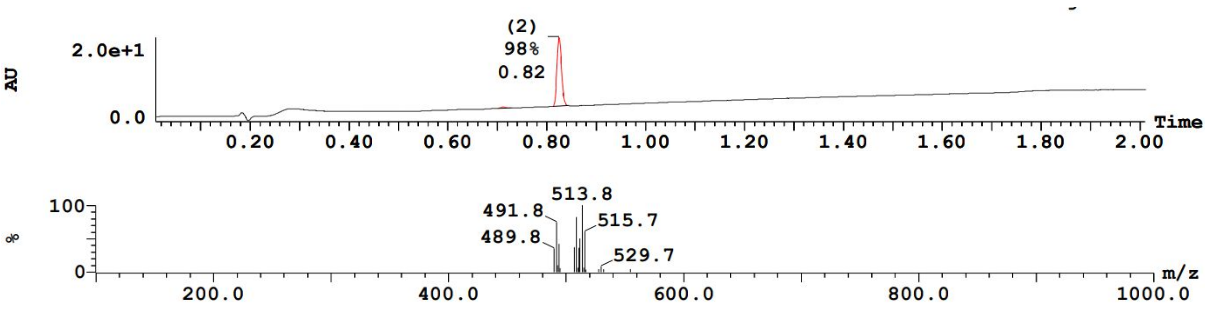

LCMS of S**7**.

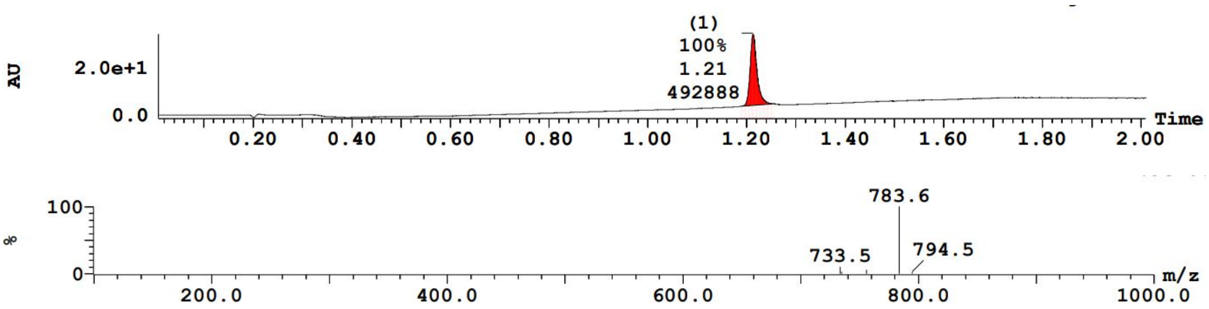

LCMS of S**8**.

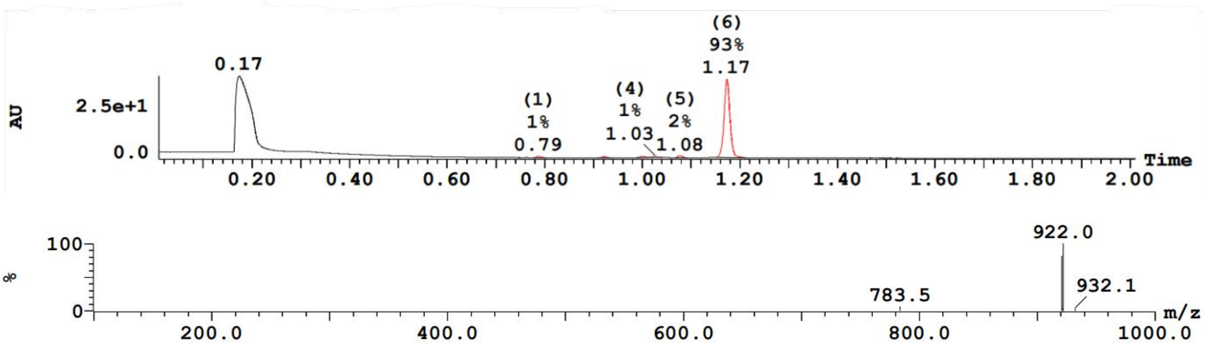

LCMS of S**10**.

1.7 NMR Spectra

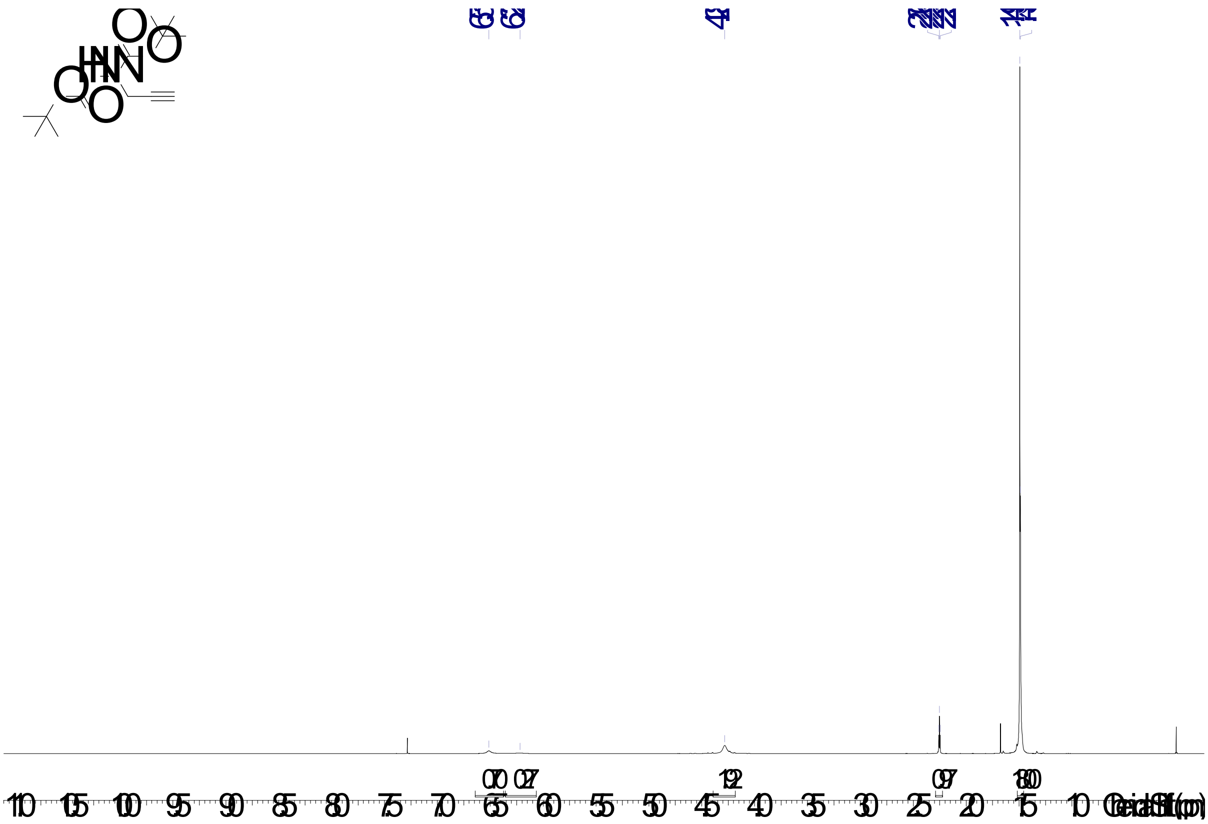

H NMR of S1.

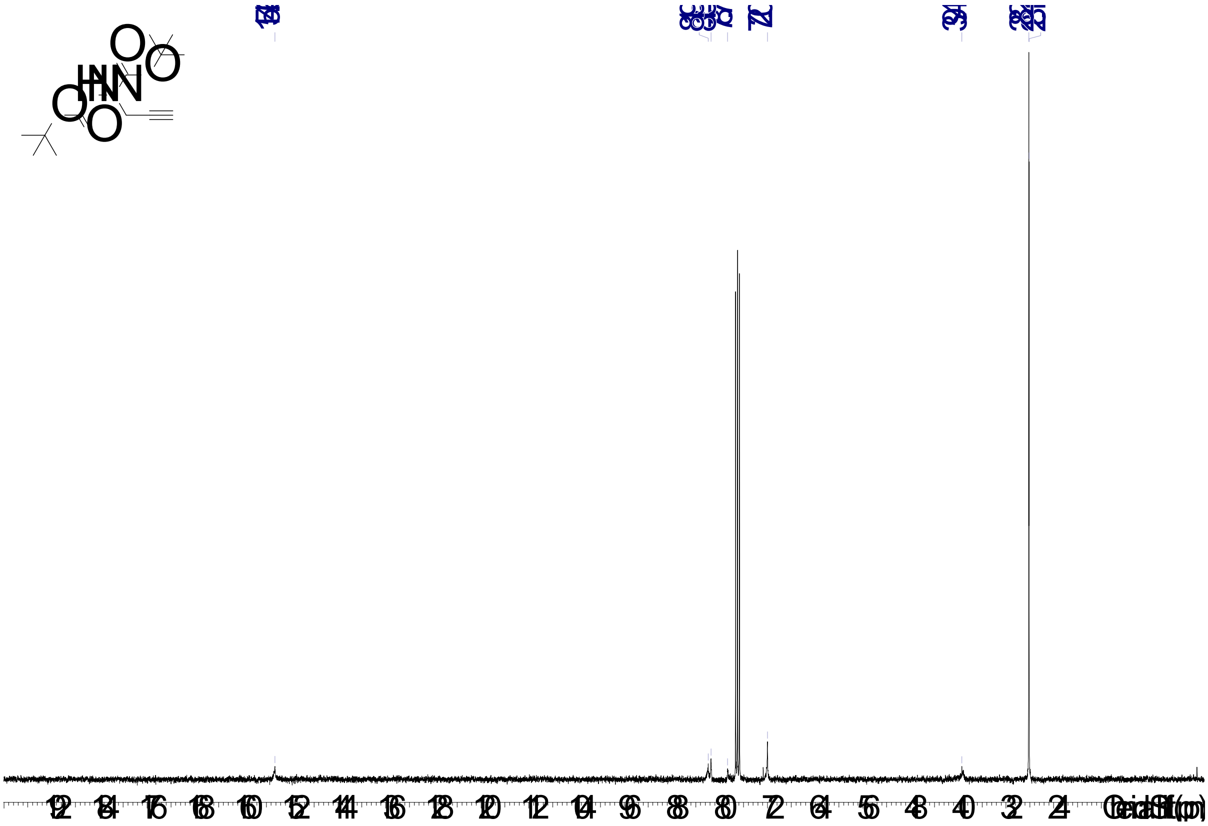

C NMR of S1.

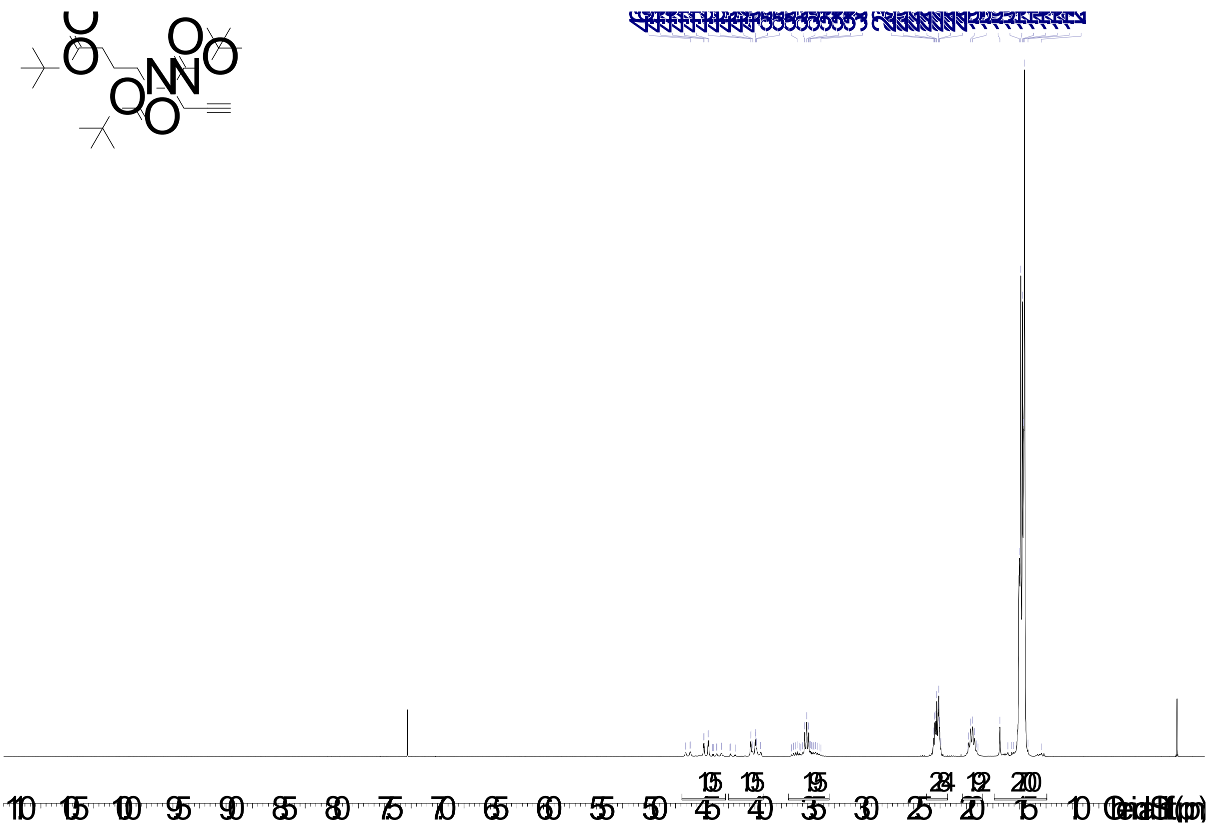

H NMR of S2.

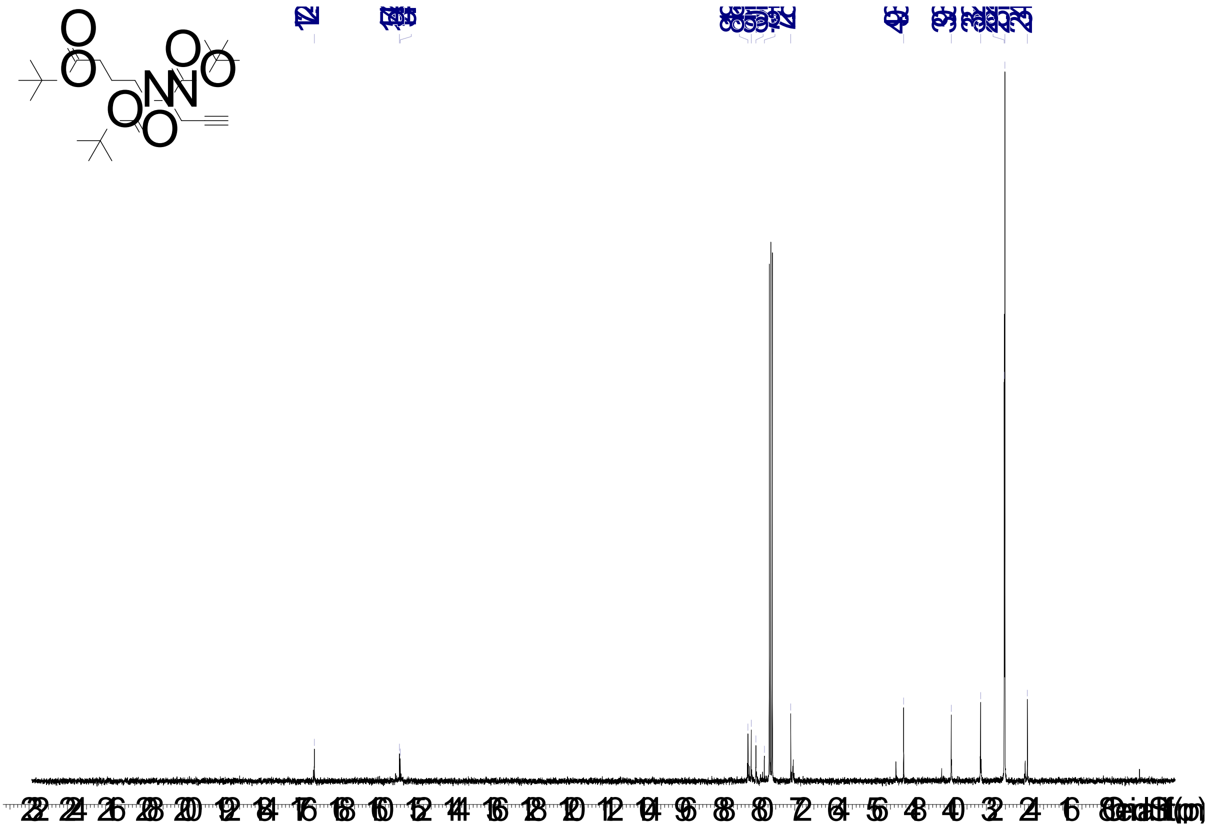

C NMR of S2.

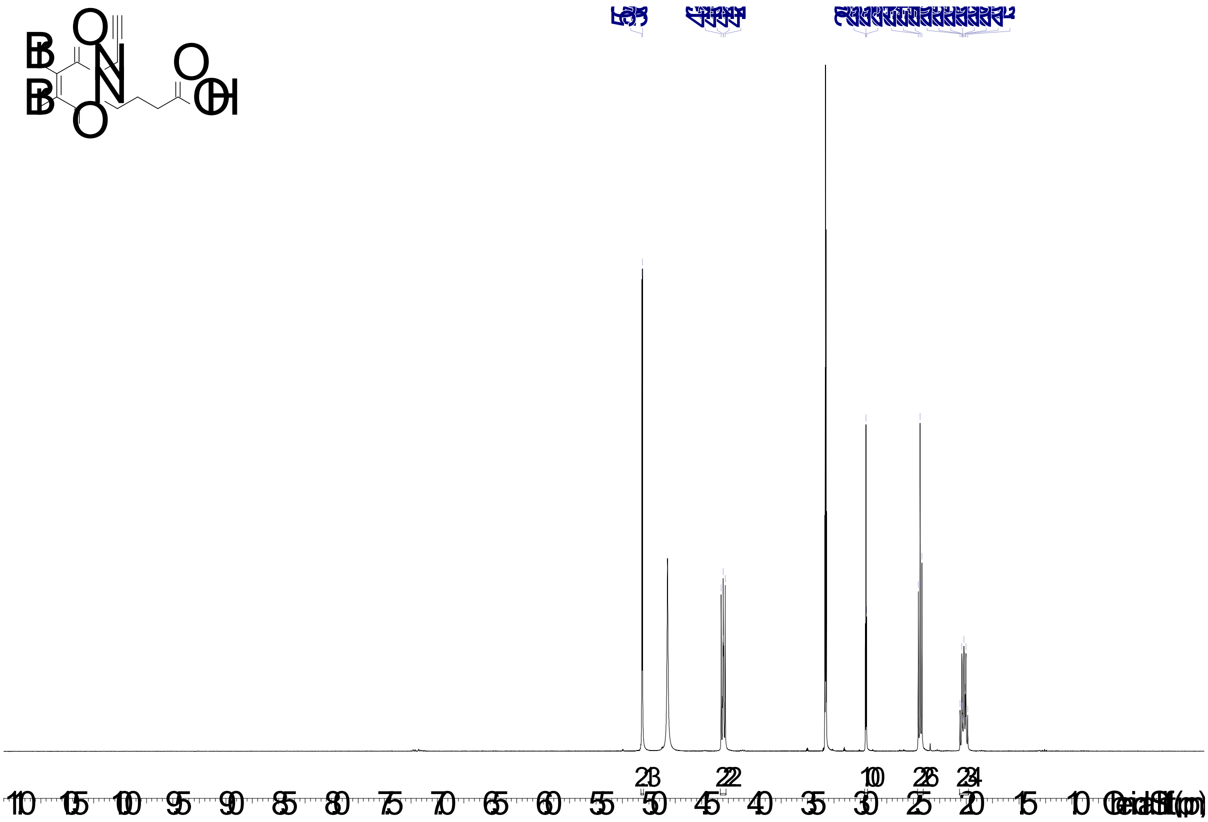

H NMR of S3.

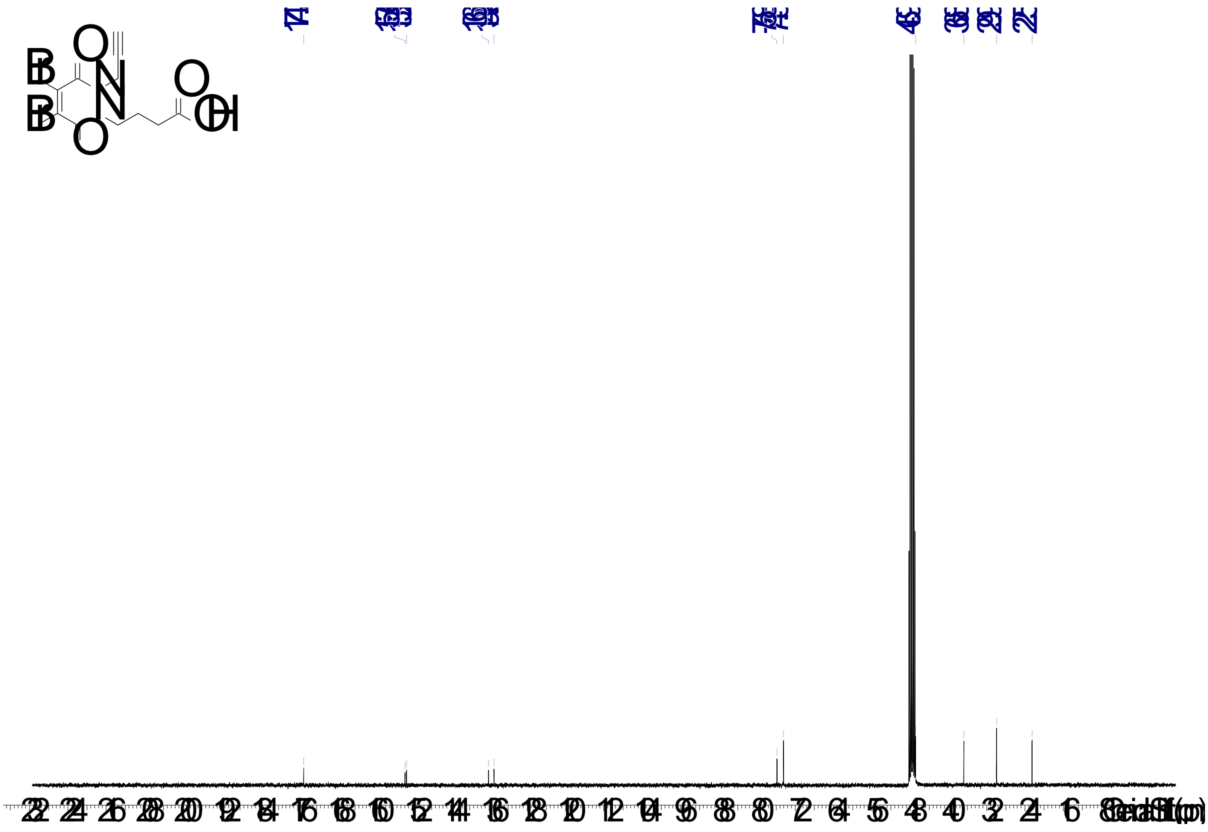

C NMR of S3.

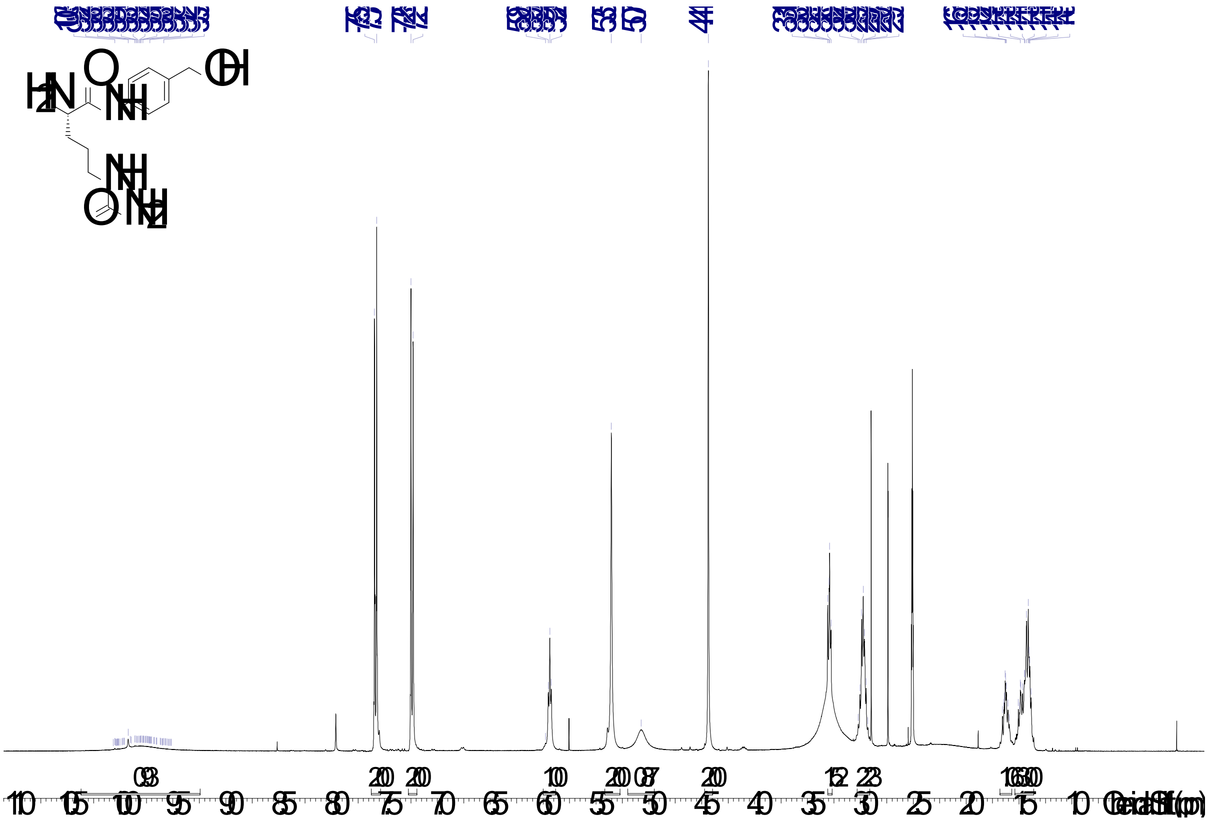

H NMR of S5.

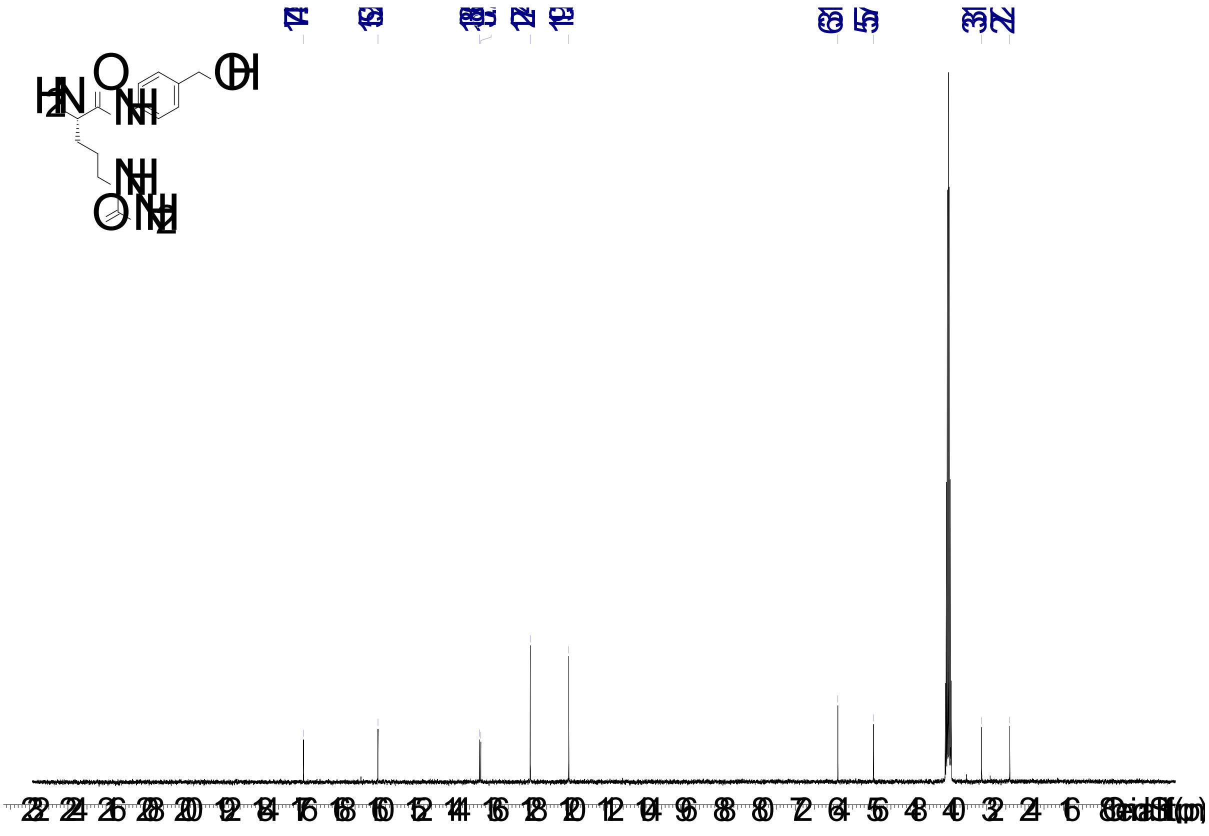

C NMR of S5.

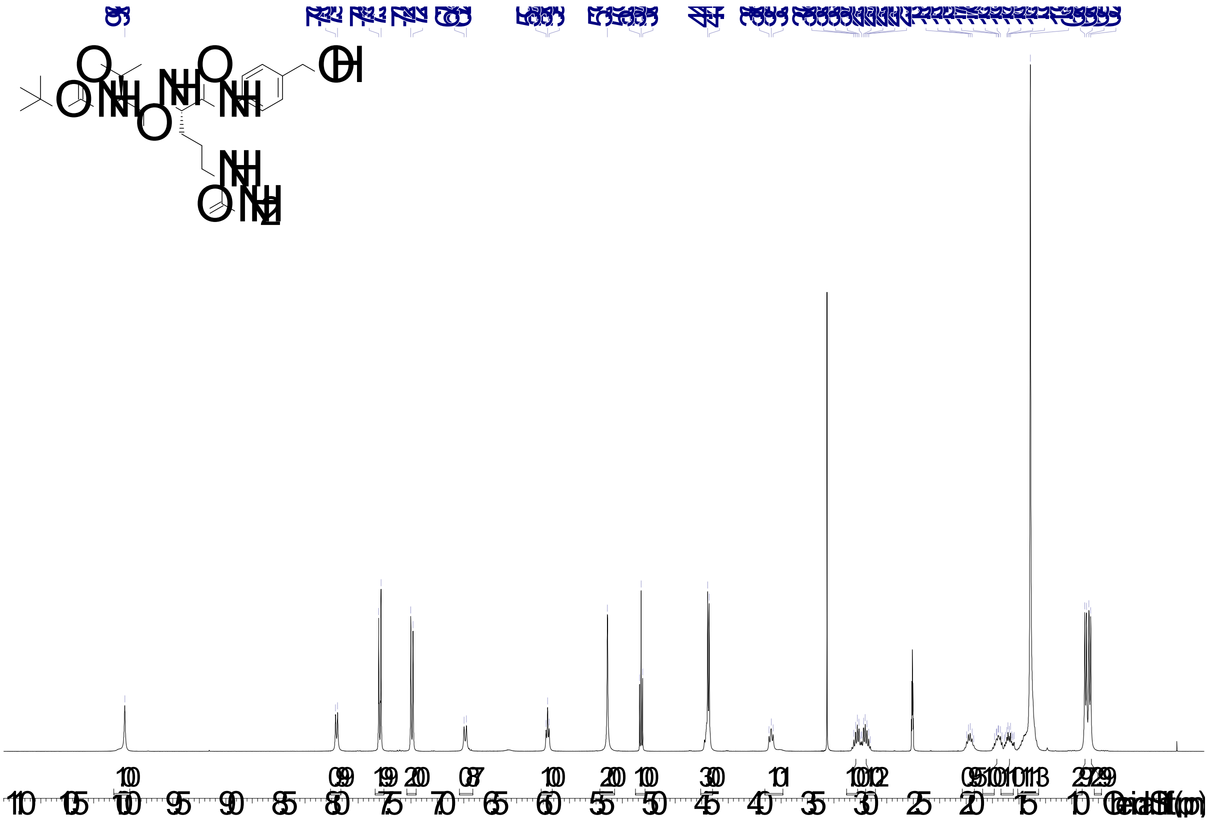

H NMR of S6.

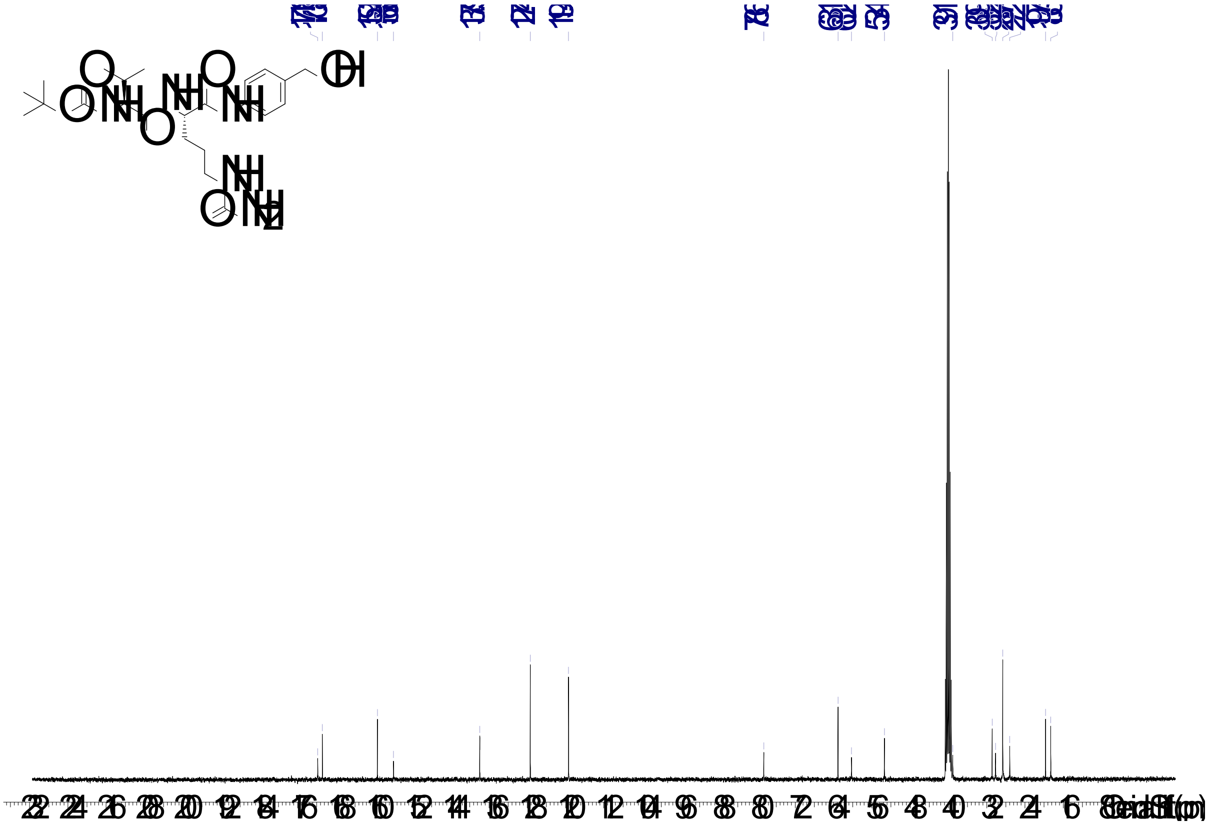

C NMR of S6.

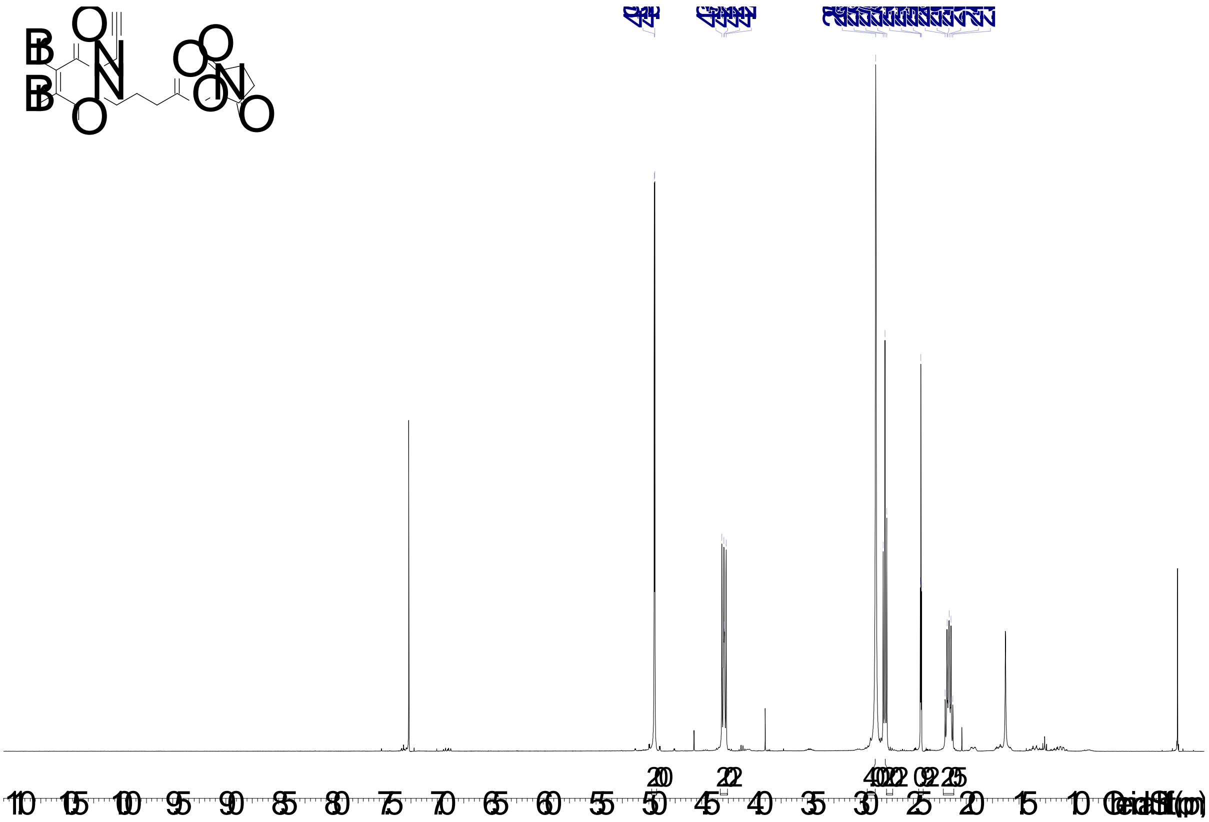

H NMR of S7.

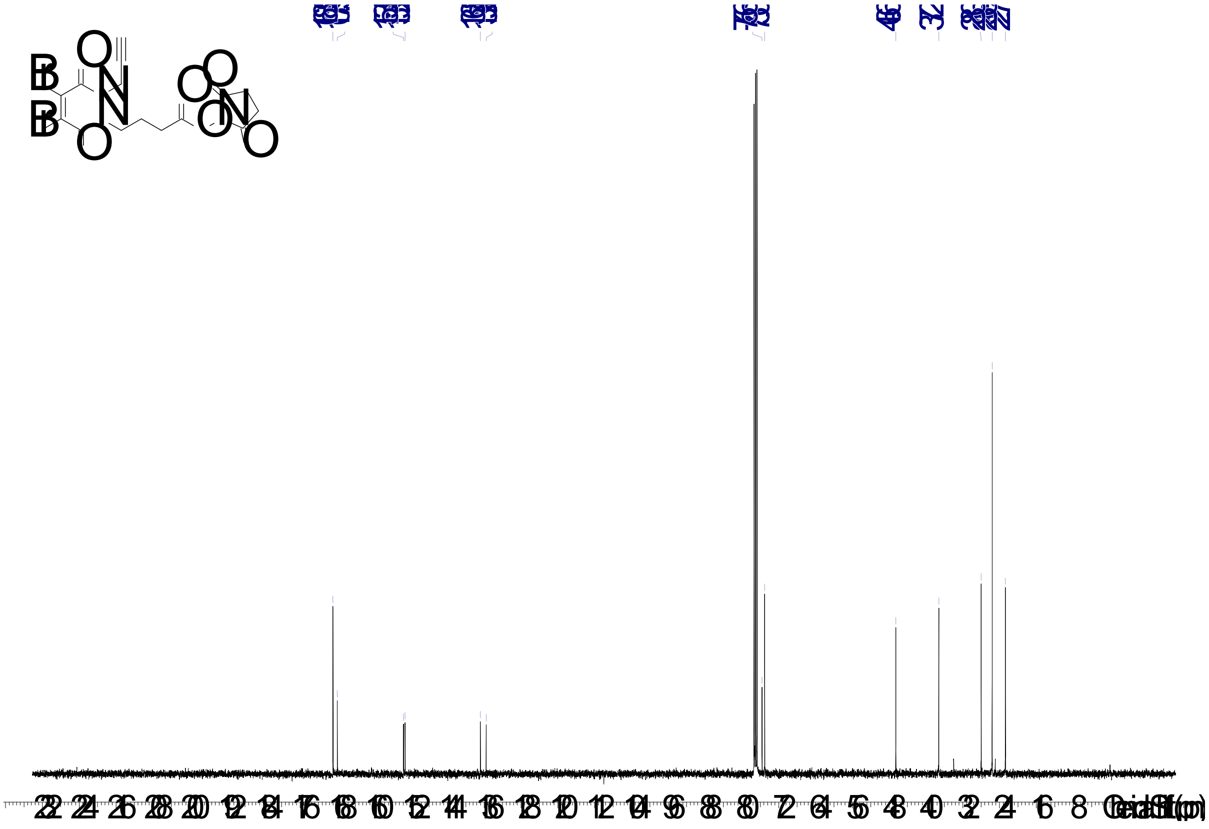

C NMR of S7.

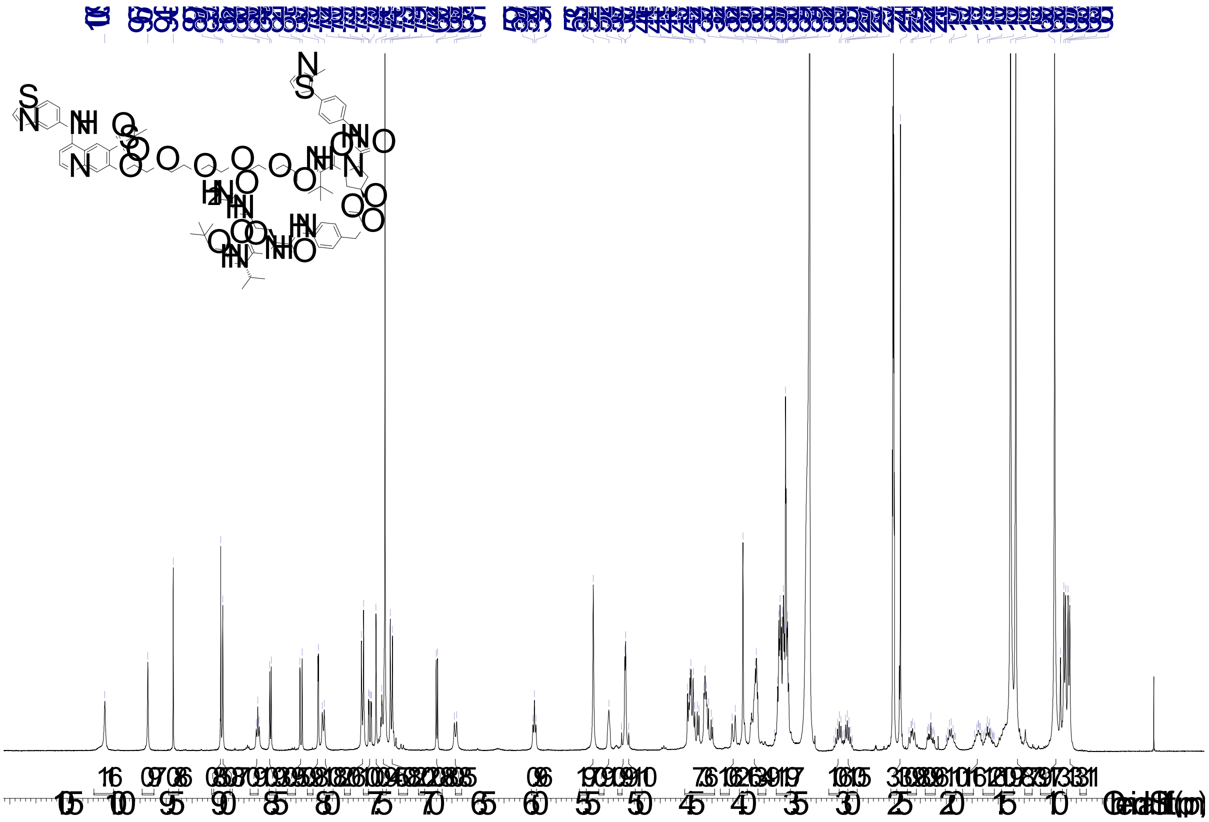

H NMR of S8.

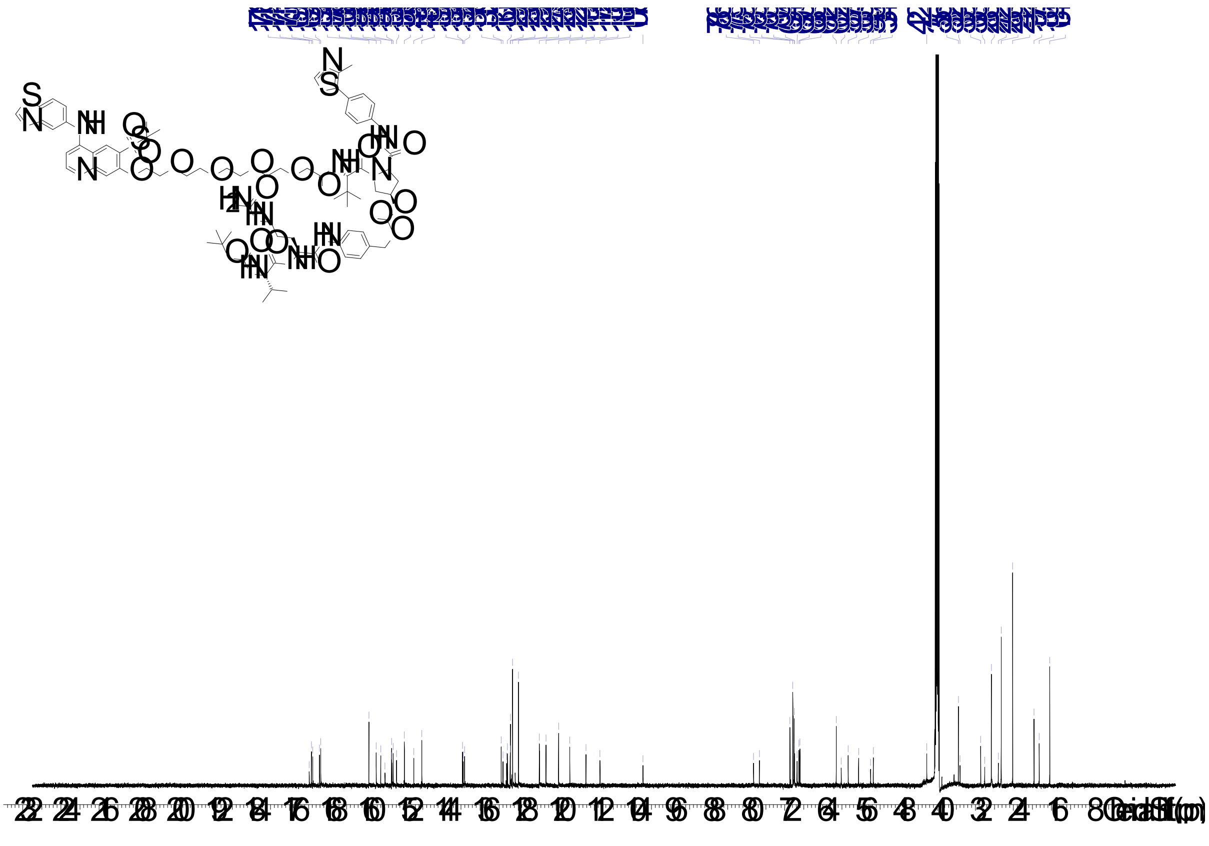

C NMR of S8.

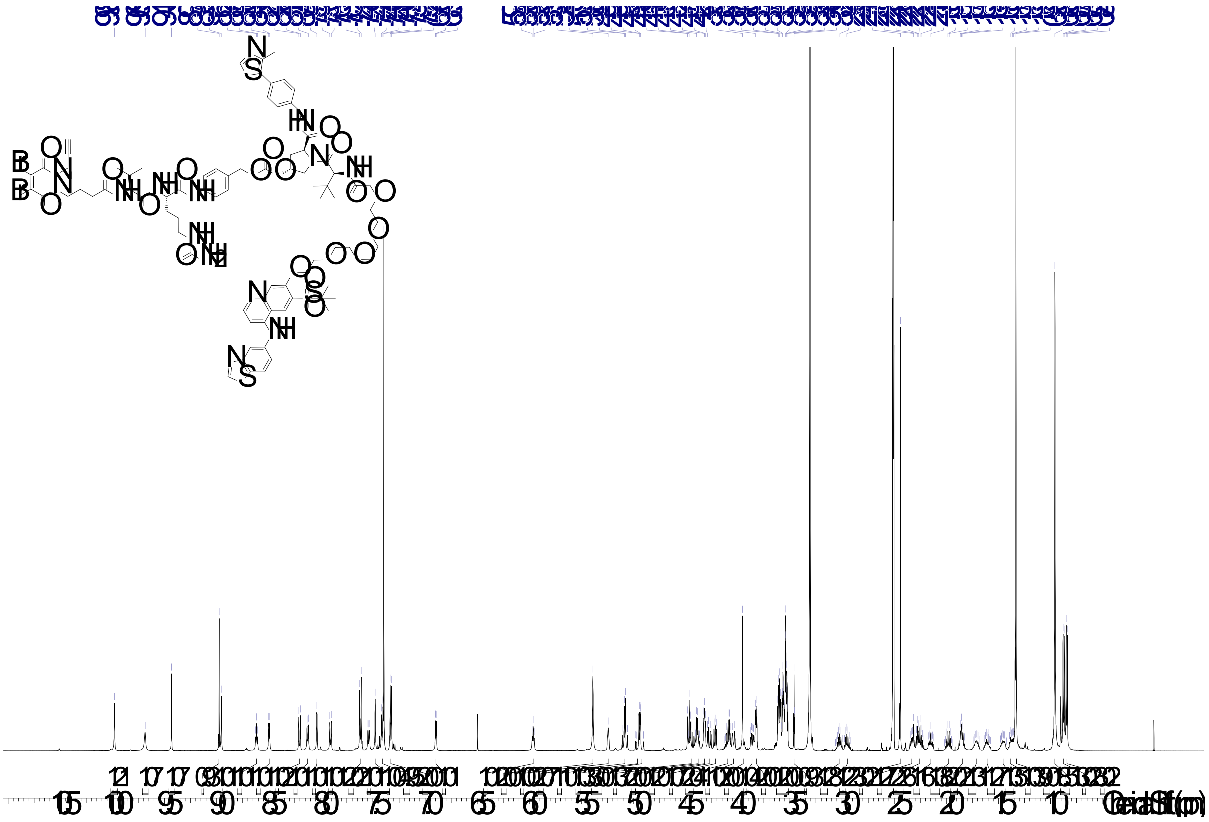

H NMR of S10.

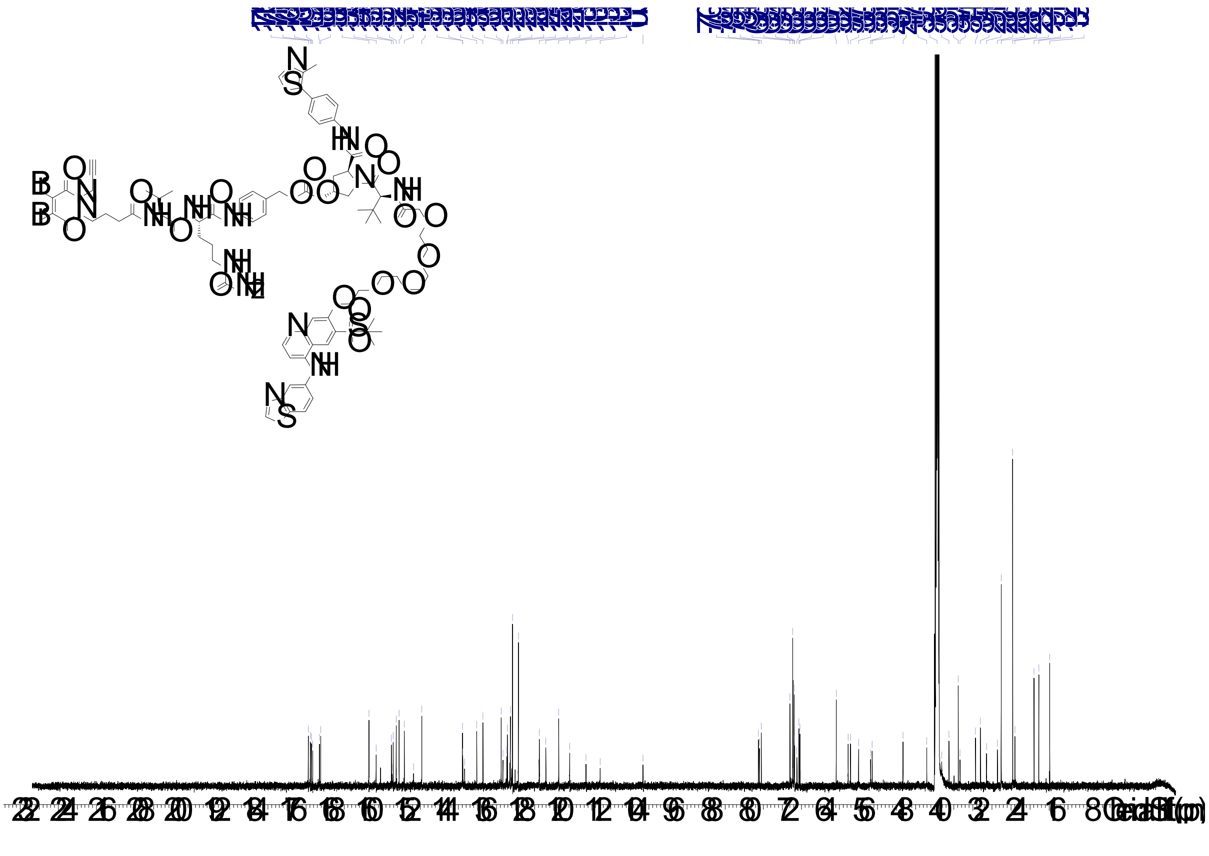

C NMR of S10.

2. Biology and Bioconjugation

2.1 Materials and methods

Solvents and reagents were purchased from commercial suppliers and used as received. Phosphate-buffered saline (PBS): 2.67 mM KCl, 1.5 mM KH_2PO_4, 138 mM NaCl, Na_2HPO_4-$7H_2O$. Borate-buffered saline (BBS): 50 mM Boric acid, 50 mM NaOH, 50 mM NaCl, 5 mM EDTA, adjusted to pH 8.5 with HCl. Conjugation experiments were carried out in standard polypropylene Eppendorf safe-lock tubes (1.5 mL) at atmospheric pressure and the temperature stated.

Centrifugation

Centrifugation was carried out in either an Eppendorf 5417R centrifuge or a Sorvall Legend XTR centrifuge. Ultrafiltration was carried out using either Merck Millipore Amicon (30,000 Da membrane) or VivaSpin 5000 concentrators (10,000 or 30,000 Da MwCO).

Protein concentration

Protein concentration was determined by measuring the absorbance at λ = 280 nm using a NanoDrop 1000 spectrophotometer.

Intact Mass Spectrometry (Intact MS)

Intact MS was performed using a Waters Acquity UPLC pump system connected with a TUV detector with Acquity RDa Waters Mass Spectrometer and using column: Waters BioResolve 2.1x50mm column. Mobile phase A was Water + 0.1% Formic acid , mobile phase B was MeCN + 0.1% Formic acid. The sample was run at a flow rate of 0.5 ml/min. The obtained m/z spectra was deconvoluted and analysed using the Unifi Version 1.9.4.053.

Gel Electrophoresis

SDS-PAGE was carried out using Invitrogen NuPage 4-12% Bis-Tris gels. Samples were mixed with SDS non-reducing loading buffer (NuPAGE LDS sample buffer 4x) or reducing loading buffer (NuPAGE LDS sample buffer + 0.5 M DTT in a 9:1 ratio). Reduced samples were heated at 90 °C for 5 min before being loaded onto the gel. Samples were run at a constant current (120 mA) and voltage (200 V) for 40 min in Novex NuPage MES SDS running buffer (20x). Gels were stained with InstantBlue® Coomassie protein stain and de-stained with H_2O. The molecular ladder used was either the SeeBlue Plus 2 pre-stained protein standard or Novex Sharp pre-stained protein standard.

Gel imaging

Gel imagery was obtained using a BioRad GeldocTM EZ Imager (White Light Sample Tray) and processed using Image Lab: Exposure Time (sec) 0.273 (Auto - Intense Bands), Application Instant Blue,

Dark Type Referenced, Ref. Bkgd. Time (sec) 10, Flat Field Applied, Serial Number 735BR07211, Software Version 6.1.0.07, Illumination Mode White Transillumination.

2.2 Expression of mAbs

mAbs were generated from HEK293 cells transfected with 1 mg of mAb DNA (0.5 mg HC + 0.5 mg LC). The media consisted of BalanCD, GlutaMAX, geneticin and 1 mg of mAb in 100 mL of OptiMEM (HEPES, 2.4 g/L sodium bicarbonate, L-Gln). The total volume was 1 L with a cell concentration was 1.58 × 10^6 cells/mL. The mixture was incubated at 37 °C for 6 days on a shaking platform at 125 rpm and 5% CO_2. After 48 h, cells were treated with tryptone (25 mL). After 72 h, cells were treated with 3 M fructose (33 mL). After 6 days, the mixture was spun at 4000 rpm for 15 min and the supernatant filtered through a Nalgene Rapid-Flow 90 mm Filter Unit. The filtrate was purified using a Protein A column and ASEC. Fractions containing product were pooled by centrifugation (30,000 Da) and buffer-exchanged into PBS. The product was filtered through a 0.2-μm filter under sterile conditions and frozen for storage.

<u>Anti-HER2 mAb sequence:</u>[3]

<u>Heavy Chain</u>

EVQLVESGGGLVQPGGSLRLSCAASGFNIKDTYIHWVRQAPGKGLEWVARIYPTNGYTRYADSVKGRFTISADTSK
NTAYLQMNSLRAEDTAVYYCSRWGGDGFYAMDYWGQGTLVTVSSASTKGPSVFPLAPSSKSTSGGTAALGCLVK
DYFPEPVTVSWNSGALTSGVHTFPAVLQSSGLYSLSSVVTVPSSSLGTQTYICNVNHKPSNTKVDKKVEPKSCDKTH
TCPPCPAPELLGGPSVFLFPPKPKDTLMISRTPEVTCVVVDVSHEDPEVKFNWYVDGVEVHNAKTKPREEQYNSTY
RVVSVLTVLHQDWLNGKEYKCKVSNKALPAPIEKTISKAKGQPREPQVYTLPPSREEMTKNQVSLTCLVKGFYPSDI
AVEWESNGQPENNYKTTPPVLDSDGSFFLYSKLTVDKSRWQQGNVFSCSVMHEALHNHYTQKSLSLSPGK

<u>Light Chain</u>

DIQMTQSPSSLSASVGDRVTITCRASQDVNTAVAWYQQKPGKAPKLLIYSASFLYSGVPSRFSGSRSGTDFTLTISSL
QPEDFATYYCQQHYTTPPTFGQGTKVEIKRTVAAPSVFIFPPSDEQLKSGTASVVCLLNNFYPREAKVQWKVDNAL
QSGNSQESVTEQDSKDSTYSLSSTLTLSKADYEKHKVYACEVTHQGLSSPVTKSFNRGEC

Anti-IL4 mAb sequence:[4]

Heavy Chain

QVTLRESGPALVKPTQTLTLTCTFSGFSLSTSGMGVSWIRQPPGKGLEWLAHIYWDDDKRYNPSLKSRLTISKDTSR
NQVVLTMTNMDPVDTATYYCARRETVFYWYFDVWGRGTPVTVSSASTKGPSVFPLAPSSKSTSGGTAALGCLVK
DYFPEPVTVSWNSGALTSGVHTFPAVLQSSGLYSLSSVVTVPSSSLGTQTYICNVNHKPSNTKVDKRVEPKSCDKTH
TCPPCPAPELLGGPSVFLFPPKPKDTLMISRTPEVTCVVVDVSHEDPEVKFNWYVDGVEVHNAKTKPREEQYNSTY
RVVSVLTVLHQDWLNGKEYKCKVSNKALPAPIEKTISKAKGQPREPQVYTLPPSREEMTKNQVSLTCLVKGFYPSDI
AVEWESNGQPENNYKTTPPVLDSDGSFFLYSKLTVDKSRWQQGNVFSCSVMHEALHNHYTQKSLSLSPGK

Light Chain

DIVLTQSPSSLSASVGDRVTITCKASQSVDYDGDSYMNWYQQKPGKAPKLLIYAASNLESGIPSRFSGSGSGTDFTF
TISSLQPEDIATYYCQQSNEDPPTFGQGTKVEIKRTVAAPSVFIFPPSDEQLKSGTASVVCLLNNFYPREAKVQWKVD
NALQSGNSQESVTEQDSKDSTYSLSSTLTLSKADYEKHKVYACEVTHQGLSSPVTKSFNRGEC

2.3 Synthesis of ADC-**2** and ADC-**3**

Anti-HER2 ADC-2

To anti-HER2 mAb (780 µL, 3.85 mg/mL, 1 equiv.) in BBS was added TCEP·HCl (20 µL, 10 mM in BBS, 10 equiv.) and the mixture incubated at 37 °C for 1.5 h. The reaction mixture was then cooled to 4 °C and to this was added diBrPD **S10** (80 µL, 10 mM in DMF, 40 equiv.) and DMF (120 µL), and the resulting mixture left to stand at 4 °C for 20 h. The excess reagents were removed *via* ultrafiltration (30,000 Da) into PBS. The conjugates were characterised by Intact MS and SDS-PAGE to determine DAR and aggregation.

Anti-IL4 ADC-3

To anti-IL4 mAb (960 µL, 3.16 mg/mL, 1 equiv.) in BBS was added diBrPD **S10** (250 µL, 1.6 mM in DMF, 20 equiv.) and the resulting mixture left to stand at 4 °C for 2 h. After the preincubation period, TCEP·HCl (20 µL, 10 mM in H_2O, 10 equiv.) was added and the resulting mixture was left to stand at 4 °C for 20 h. The excess reagents were removed *via* ultrafiltration (30,000 Da) into PBS. The conjugates were characterised by Intact MS and SDS-PAGE to determine DAR and aggregation.

3. Biological Assays

3.1 Materials and Methods

Cell culture

SKOV-3 warranted ovarian cancer cells were cultured in McCoy's 5A medium supplemented with glutamine, 10% FBS and 1% Penicillin-Streptomycin. HEK293 cells were cultured in Dulbecco's modified Eagle's medium (DMEM) supplemented with glutamax, pyruvate, 10% heat inactivated FBS and 1% Penicillin-Streptomycin. Cell lines were maintained in a humidified incubator at 37 °C and 5% CO_2.

For cellular degradation, 4×10^6 cells were seeded in a 96-well plate, allowed to attach overnight, and incubated at 37 °C for 6 h or 16 h with the indicated compounds. Where indicated, a 1 h pre-treatment with 10 µM MG132 was performed before the addition of the compound.

Lysis buffer composition

The lysis buffer used consists of 10 mL RIPA buffer, 1 µL of 1 M DTT, one PhosSTOP™ phosphatase inhibitor tablet, one Pierce™ protease inhibitor tablet and 25 µL Benzonase Nuclease (Sigma Aldrich).

Gel electrophoresis

SDS-PAGE was carried out using Invitrogen NuPage 4-12% 1.5 mm Bis-Tris gels. Samples were mixed with loading buffer (9:1 ratio of NuPAGE LDS sample buffer 4x/NuPAGE sample reducing agent 10x) and then heated at 95 °C for 5 min. Samples were run at a constant voltage (200 V) for 60 min in Novex NuPage MOPS SDS running buffer (20x). The molecular ladder used was the Li-COR Chameleon Duo Pre-stained ladder.

Western blot analysis

After cell treatment, the media was aspirated and to each well was added 25-30 µL of lysis buffer. The cells were left on a rocker at 4 °C for 20 min before subjecting the protein extracts to SDS-PAGE. Each gel was subjected to wet transfer on low background fluorescence PVDF membranes which were then blocked with LI-COR Intercept® Blocking Buffer for 1 h at rt. The membranes were then incubated with the primary antibodies at 4 °C overnight, followed by PBS + 0.1% tween washes (3×10 min), and then incubation with the secondary antibodies for 1 h at rt (see Table S1 for antibodies). The membranes were washes with PBS + 0.1% tween (3×5 min) and then visualised using the Odyssey LCx imaging system and analysed using ImageStudio Lite Version 5.2.

Table S1. Primary and secondary antibodies used.

Antibody	Species	Supplier	Catalog no.	Dilution
RIPK2	Rabbit	Cell Signalling Technologies	4142S	1:1000
β-actin	Mouse	Cell Signalling Technologies	3700S	1:1000
800CW Anti-rabbit IgG	Donkey	LI-COR	926-32213	1:20000
680RD Anti-mouse IgG	Donkey	LI-COR	926-68072	1:20000

HiBit Assay

The Promega Nano-Glo® HiBit Lytic Detection System was used for analysis according to the manufacturer's instructions. The plate was read on a PHERAstar.

Cell viability

Cell viability was determined by CellTiter-Glo luminescent cell viability assay according to the manufacturer's instructions (Promega, G7570).

3.2 Uncropped blots

SKOV3 16 h incubation

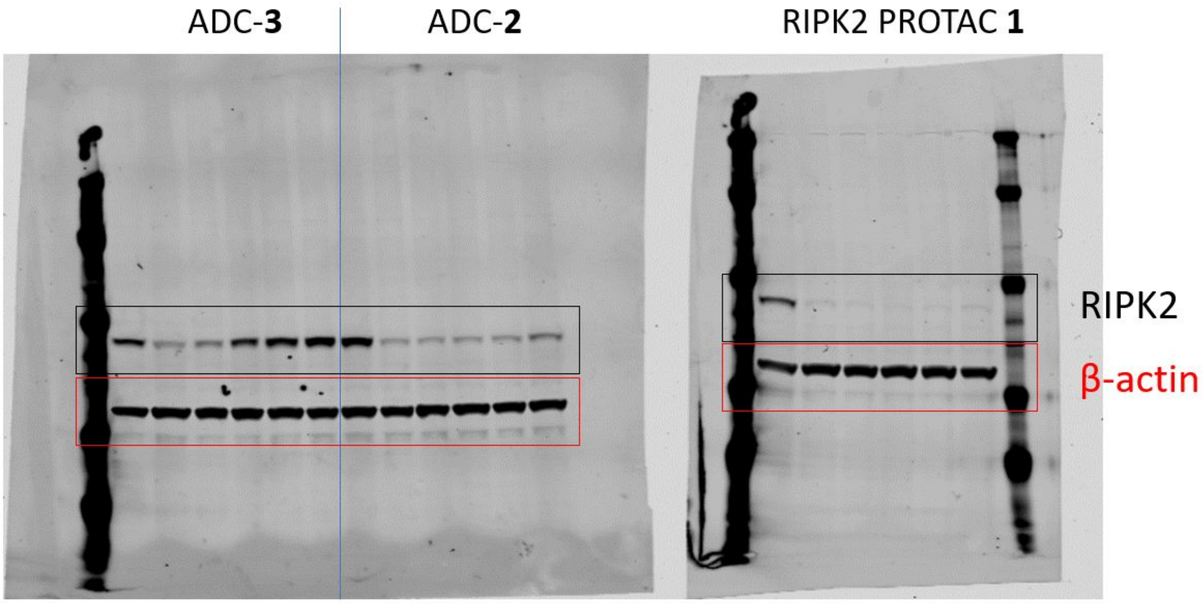

Figure S10. Uncropped Western blot analysis of RIPK2 degradation in SKOV3 cells following a 16 h incubation with PROTAC **1**, ADC-**2** or ADC-**3**.

SKOV3 6 h incubation

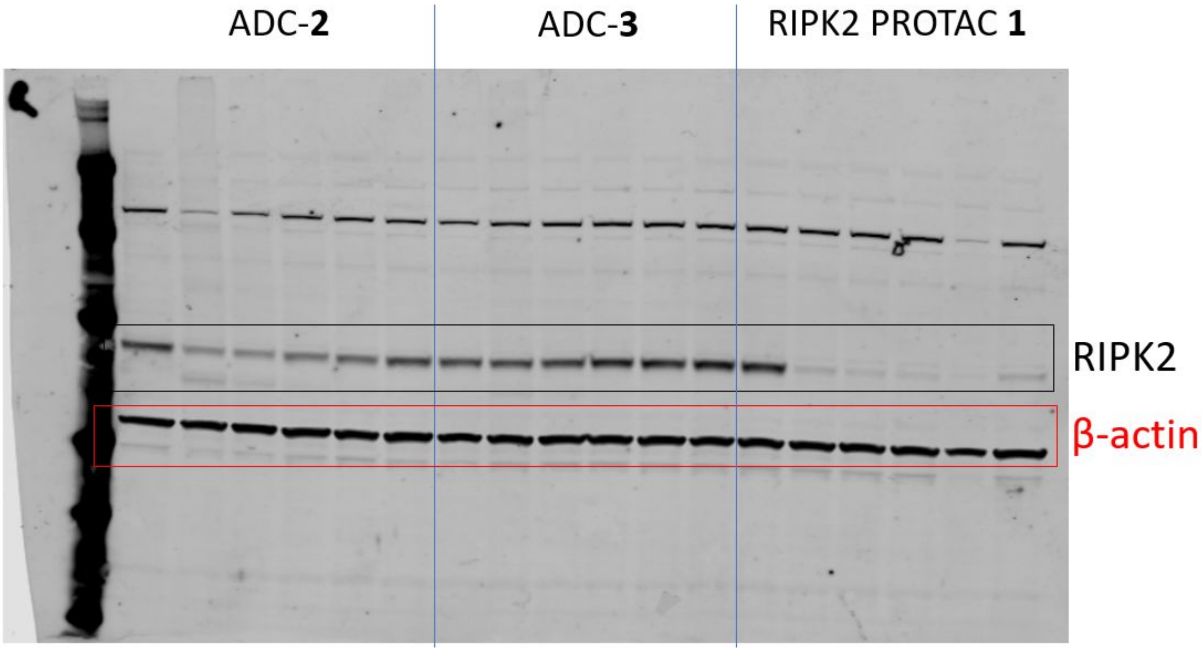

Figure S11. Uncropped Western blot analysis of RIPK2 degradation in SKOV3 cells following a 6 h incubation with PROTAC **1**, ADC-**2** or ADC-**3**.

SKOV3 + MG132

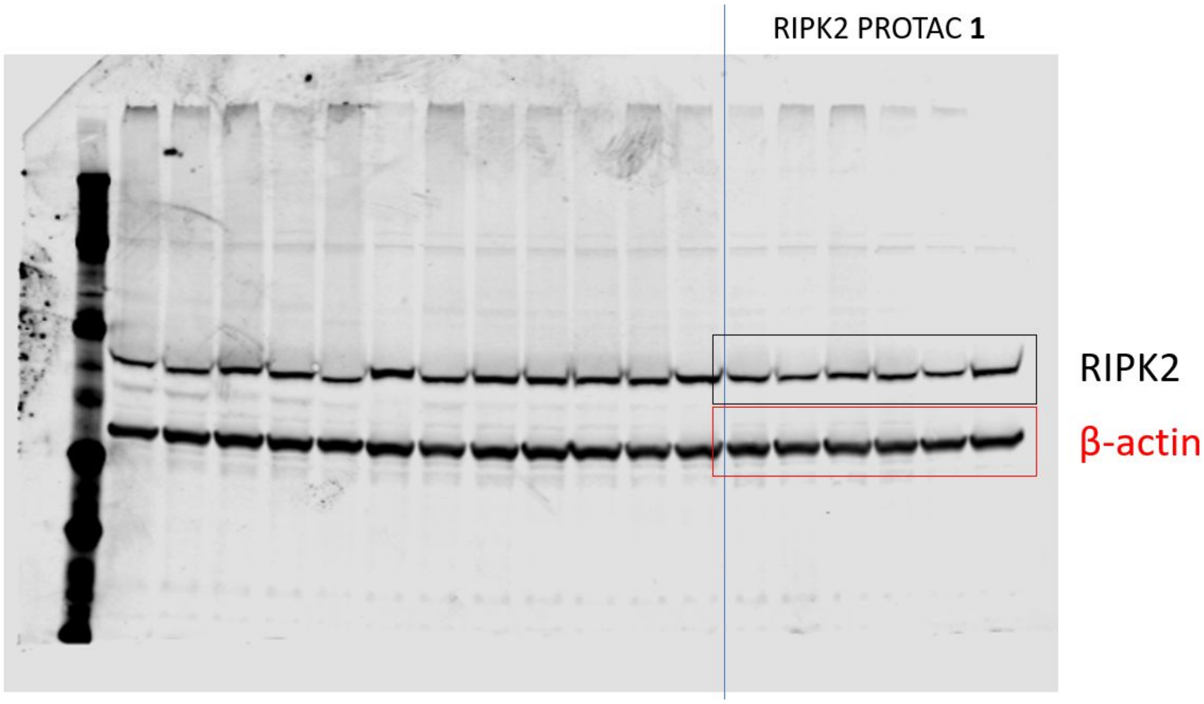

Figure S12. Uncropped Western blot analysis of RIPK2 degradation in SKOV3 cells following a 1 h pre-treatment with 10 μM MG132 following a 16 h incubation with PROTAC **1**.

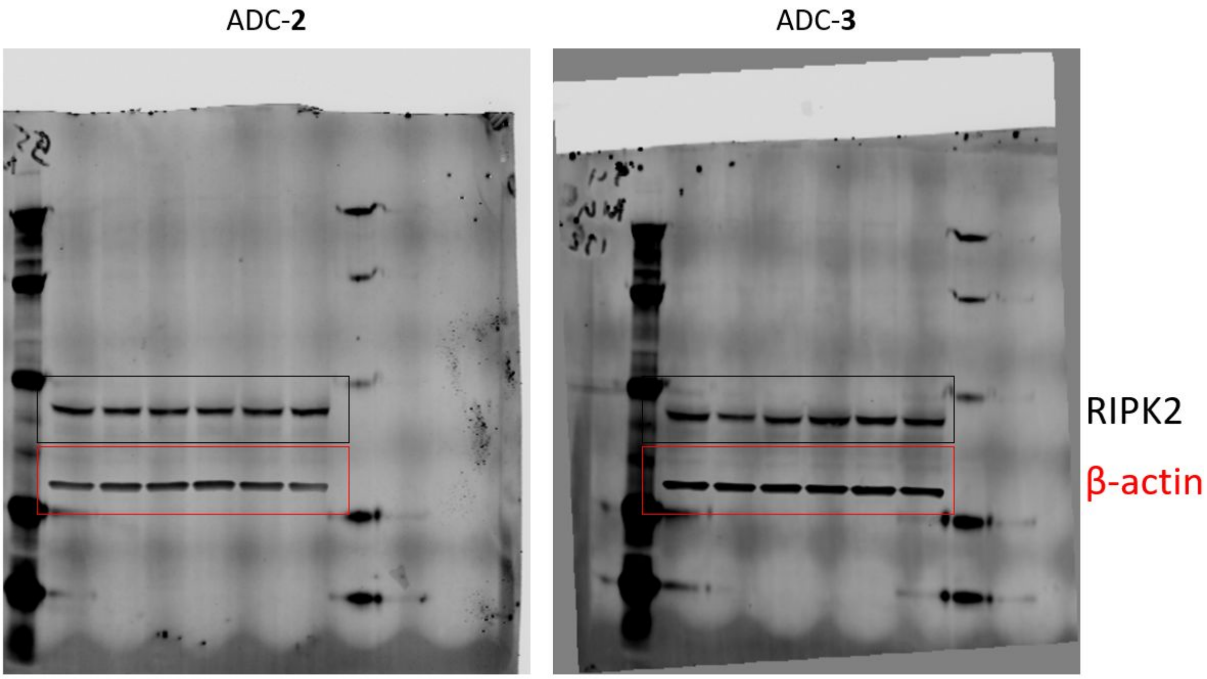

Figure S13. Uncropped Western blot analysis of RIPK2 degradation in SKOV3 cells following a 1 h pre-treatment with 10 µM MG132 following a 16 h incubation with ADC-**2** or ADC-**3**.

4. References

1. Bondeson, D. P.; Mares, A.; Smith, I. E.; Ko, E.; Campos, S.; Miah, A. H.; Mulholland, K. E.; Routly, N.; Buckley, D. L.; Gustafson, J. L.; Zinn, N.; Grandi, P.; Shimamura, S.; Bergamini, G.; Faelth-Savitski, M.; Bantscheff, M.; Cox, C.; Gordon, D. A.; Willard, R. R.; Flanagan, J. J.; Casillas, L. N.; Votta, B. J.; den Besten, W.; Famm, K.; Kruidenier, L.; Carter, P. S.; Harling, J. D.; Churcher, I.; Crews, C. M., Catalytic in vivo protein knockdown by small-molecule PROTACs. *Nat. Chem. Biol.* **2015,** *11*, 611-617.
2. Marcher, A.; Nijenhuis, M. A. D.; Gothelf, K. V., A Wireframe DNA Cube: Antibody Conjugate for Targeted Delivery of Multiple Copies of Monomethyl Auristatin E. *Angew. Chem., Int. Ed. Engl.* **2021,** *60*, 21691-21696.
3. National Center for Advancing Translational Sciences: *Trastuzumab*. https://drugs.ncats.io/drug/P188ANX8CK (accessed 2023).
4. National Center for Advancing Translational Sciences: *Pascolizumab*. https://drugs.ncats.io/substance/N1IOA09R6A (accessed 2023).
